# Polypropylene nanoplastic exposure leads to lung inflammation through p38-mediated NF-κB pathway due to mitochondrial damage

**DOI:** 10.1186/s12989-022-00512-8

**Published:** 2023-01-10

**Authors:** Jong-Hwan Woo, Hyeon Jin Seo, Jun-Young Lee, Iljung Lee, Kisoo Jeon, Bumseok Kim, Kyuhong Lee

**Affiliations:** 1 Inhalation Toxicology Center for Airborne Risk Factor, Korea Institute of Toxicology, 30 Baehak1-gil, Jeongeup, Jeollabuk-do 56212 Republic of Korea; 2 Biosafety Research Institute and Laboratory of Pathology, College of Veterinary Medicine, Jeonbuk National University, Iksan-Si, Jeollabuk-do Republic of Korea; 3 Department of Human and Environmental Toxicology, University of Science and Technology, Daejeon, 34113 Republic of Korea; 4 Korea Radioisotope Center for Pharmaceuticals, Korea Radiological and Medical Sciences, 75 Nowon-ro, Nowon-gu, Seoul, 01812 Republic of Korea; 5 VITALS Co., Ltd., 30 Gukjegwahak 3-ro, Yuseong-hu, Daejeon, 34000 Republic of Korea

**Keywords:** Polypropylene (PP), Nanoplastic, Microplastic, Mitochondrial damage, p38, NF-κB

## Abstract

**Background:**

Polypropylene (PP) is used in various products such as disposable containers, spoons, and automobile parts. The disposable masks used for COVID-19 prevention mainly comprise PP, and the disposal of such masks is concerning because of the potential environmental pollution. Recent reports have suggested that weathered PP microparticles can be inhaled, however, the inhalation toxicology of PP microparticles is poorly understood.

**Results:**

Inflammatory cell numbers, reactive oxygen species (ROS) production, and the levels of inflammatory cytokines and chemokines in PP-instilled mice (2.5 or 5 mg/kg) increased significantly compared to with those in the control. Histopathological analysis of the lung tissue of PP-stimulated mice revealed lung injuries, including the infiltration of inflammatory cells into the perivascular/parenchymal space, alveolar epithelial hyperplasia, and foamy macrophage aggregates. The in vitro study indicated that PP stimulation causes mitochondrial dysfunction including mitochondrial depolarization and decreased adenosine triphosphate (ATP) levels. PP stimulation led to cytotoxicity, ROS production, increase of inflammatory cytokines, and cell deaths in A549 cells. The results showed that PP stimulation increased the p-p38 and p-NF-κB protein levels both in vivo and in vitro, while p-ERK and p-JNK remained unchanged. Interestingly, the cytotoxicity that was induced by PP exposure was regulated by p38 and ROS inhibition in A549 cells.

**Conclusions:**

These results suggest that PP stimulation may contribute to inflammation pathogenesis via the p38 phosphorylation-mediated NF-κB pathway as a result of mitochondrial damage.

**Supplementary Information:**

The online version contains supplementary material available at 10.1186/s12989-022-00512-8.

## Background

Worldwide, plastics are used in various products, such as face masks, disposable products, plumbing, toys, and automobile parts [1, 2]. With the increasing use of plastic-based products, the likelihood of human exposure to microplastics has increased. In addition, as preventive measures for combating the COVID-19 pandemic continue, the increasing use of disposable masks that mainly comprise polypropylene (PP) is raising concerns about environmental pollution and the potential exposure of the human body to microplastics [3–5]. The major sources of airborne microplastic particles are the manufacture, use, and weathering of plastic products. Several microplastics, such as PP, polystyrene (PS), and polyethylene terephthalate (PET) particles and fibres have been detected in the atmosphere as a result of the action of physical forces, hydrolysis, and ultraviolet radiation [6–9]. Atmospheric microplastics float from various regions around the general population, including roads, sea spray, agricultural dust, and dust near population centers [8]. These microplastics float in the air and exposure to the human respiratory system through the inhalation route is probably the most important exposure route to microplastics in the human body. Microplastics such as PP, polyethylene (PE), and PET have been detected in the lung tissue of live humans, with PP microplastics (particles and fibres) being the most dominant (23%) [10]. Polymer-type PP microplastics have also been detected in human lung tissue [11]. Recent studies have reported that airborne PP microplastics accumulated in lung tissue upon inhalation [5, 10–12], however, the toxic effects of PP microparticles in the respiratory system remain poorly understood.

Microplastics (< 5.00 mm), or plastics that become micronised upon weathering and exposure to sunlight can be divided into medium (1.01–5 mm), small (< 1 mm), and nano-sized microplastics (< 1 μm) [12, 13]. Nanoplastics, in particular, can easily infiltrate cells and tissues as a result of their small size, and the accumulation of these materials in organs alters physiological processes [12, 13]. Microplastics that are 1–5 μm in size can infiltrate the respiratory tract to reach the lung tissue via the lower airway, and nanosized microplastics can even infiltrate the alveolus [12]. Inhaled microplastic particles may be translocated by active cellular uptake, which occurs via contact with the bronchial epithelium after penetration of the lung lining fluid [6]. Nanosized plastic particles have been observed to infiltrate the pulmonary epithelial barrier and easily pass into the bloodstream [12]. Recent studies have reported that the inhalation of nanosized plastic can lead to bronchial epithelial injury as a result of epithelial barrier infiltration, leading to inflammatory response, cytotoxicity, and genotoxicity, and that long-term exposure can lead to pulmonary diseases such as asthma and pneumoconiosis [6, 7, 12, 14]. Although several studies have reported on the toxicity of nanoplastics, the underlying mechanisms responsible for pulmonary toxicity due to nanoplastic inhalation are still not completely understood.

Mitochondria play a key role in producing energy for use by cellular organelles [15]. In addition to supplying cellular energy, mitochondria are involved in cellular metabolism, including processes such as cellular differentiation, cell death, and growth [15, 16]. Mitochondrial damage is known to induce mitochondrial dysfunctions such as depolarisation of the mitochondrial membrane potential, a decrease in adenosine triphosphate (ATP) levels, and the production of reactive oxygen species (ROS), which are important factors in cellular metabolism and have been implicated in various human diseases, including asthma and pulmonary fibrosis [15–19]. Previous reports have suggested that the mitochondrial injury that results from exposure to airborne microparticles led to toxic responses such as oxidative stress, cytotoxicity, and inflammation through the mitogen-activated protein kinase (MAPK) and nuclear factor kappa B (NF-κB) signalling pathways [12, 20]. In addition, previous studies have reported that particulate matter (PM)-instilled mice showed oxidative stress and airway inflammatory responses due to MAPK and NF-κB activation [21]. The toxic mechanisms of these airborne particles have been reported in several in vitro and in vivo studies [12, 20–23], however, the mechanism by which mitochondrial injury occurs following exposure to toxic PP microplastics is unclear.

In this study, we characterised PP nanoplastics according to their size and shape. We also investigated the inflammatory response by analysing the inflammatory cytokine and chemokine levels, cellular changes, and histology of PP-instilled mice. We also examined the mechanisms of cytotoxicity, oxidative stress, and the inflammatory response that result from mitochondrial damage in PP-stimulated human lung epithelial cells (A549 cells).

## Results

### PP nanoplastics characterization

**Fig. 1:**
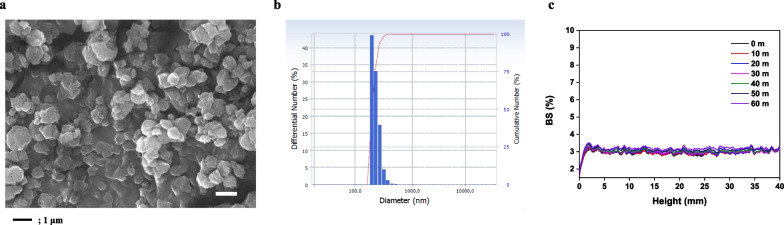
**a** FE-SEM image of PP microparticles. **b** DLS histogram showing the size distribution of PP microparticles. **c** Representative BS profiles and Turbiscan Stability Index (TSI) of PP microparticles. Scale bar 1 μm

Field emission scanning electron microscopy (FE-SEM) confirmed that the PP nanoplastics were irregular fragments with a spherical shape and 0.66 ± 0.27 μm in size (Fig. 1a). The suspension of the PP nanoplastics was analyzed using DSL, which indicated that the 252 nm particles were the most widely distributed (Fig. 1b). The dispersion stability of the PP nanoplastics suspension was measured for 1 h at 10-min intervals using a Turbiscan, which indicated that the PP nanoplastics remained stably suspended in the liquid, with little change observed in the backscattering (BS) of 0.4% for 1 h (Fig. 1c).

### PP induces inflammation and ROS production in the lung of mice

**Fig. 2:**
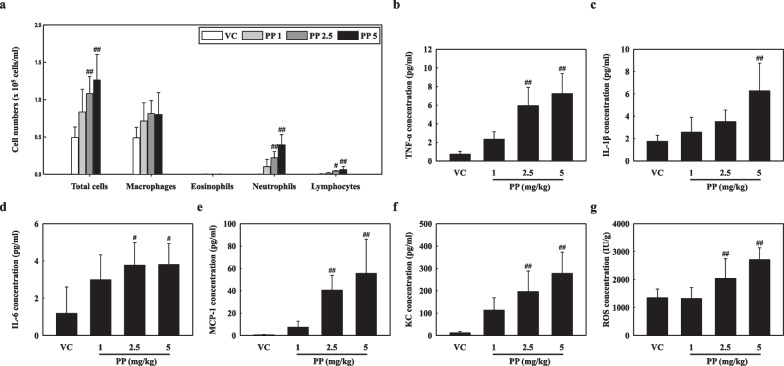
**a** Cellular changes in the BALF obtained from VC, PP 1 mg/kg (PP 1), PP 2.5 mg/kg (PP 2.5), and PP 5 mg/kg (PP 5) mice. Inflammatory cytokine and chemokine levels of BALF, including **b** TNF-α, **c** IL-1β, **d** IL-6, **e** MCP-1, and **f** KC. **g** ROS production in lung tissue. Data are presented as mean ± SD (n = 6 per group). ^#^*P* ≤ 0.05; ^##^*P* ≤ 0.01 vs. VC

**Fig. 3:**
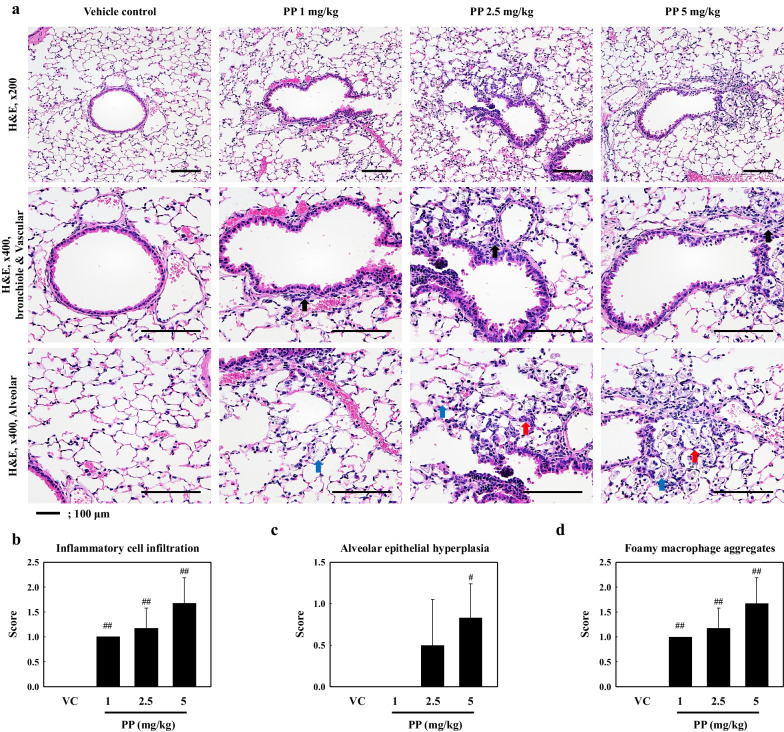
**a** Representative H&E-stained section of lung tissue. **b** Histological scoring of inflammatory cell infiltration, **c** alveolar epithelial hyperplasia, and **d** foamy macrophage aggregates. Black, red, and blue arrows indicate inflammatory cell infiltration in the perivascular/parenchymal layer, alveolar epithelial hyperplasia, and foamy macrophage aggregates, respectively. Data are presented as mean ± SD (n = 6 per group). ^#^*P* ≤ 0.05; ^##^*P* ≤ 0.01 vs. VC. Scale bar 100 μm

Toxic responses such as inflammation and ROS production were observed in the lungs of mice following PP nanoplastic stimulation. Our results showed that the number of total cells, macrophages, neutrophils, and lymphocytes in the bronchoalveolar lavage fluid (BALF) in the 2.5 and 5 mg/kg of PP-instilled mice increased significantly as compared to that in vehicle control (VC) (Fig. 2a). Additionally, the levels of inflammatory cytokines and chemokines in the BALF, including tumor necrosis factor-α (TNF-α), interleukin (IL)-1β, IL-6, monocyte chemoattractant protein-1 (MCP-1), and C-X-C motif chemokine ligand 1 (CXCL1/KC) were significantly increased in the 2.5 and/or 5 mg/kg PP intratracheal instillation groups as compared to the VC groups (Fig. 2b–f). ROS production was also increased in the lung tissue of PP-instilled mice (2.5 and 5 mg/kg) compared to the control (Fig. 2g). Histopathological analysis of the lung tissue of PP-instilled mice showed lung lesions such as inflammatory cell infiltration, alveolar epithelial hyperplasia, and foamy macrophage aggregates (Fig. 3).

### PP-instilled mice regulate p38 and NF-κB activation

**Fig. 4:**
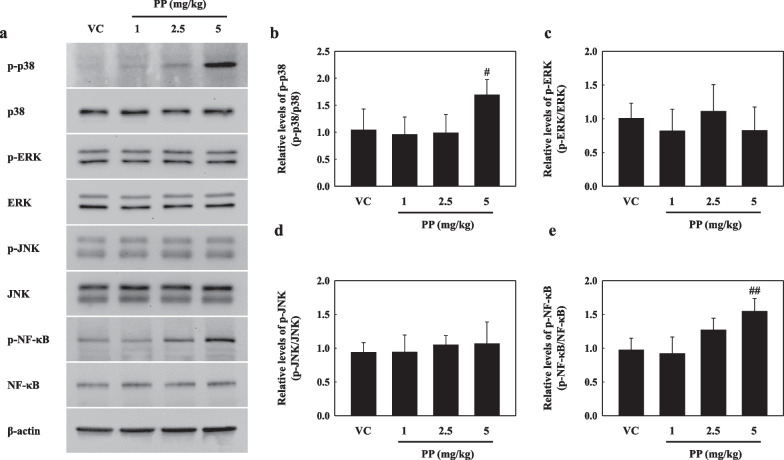
**a** Representative western blotting analysis of p-p38, p38, p-ERK, ERK, p-JNK, JNK, p-NF-κB, and NF-κB in lung tissue of PP-instilled mice. **b** Relative density analysis of p-p38 levels. Data were normalized against p38. **c** Relative density analysis of p-ERK levels. Data were normalized against ERK. **d** Relative density analysis of p-JNK levels. Data were normalized against JNK. **e** Relative density analysis of p-NF-κB levels. Data were normalized against NF-κB. Data are means ± SD (n = 6 per group). ^#^*P* ≤ 0.05; ^##^*P* ≤ 0.01 vs. VC

Our results showed that the p-p38 protein levels in the lung tissue of PP-instilled mice were significantly increased compared to those in the VC groups, while extracellular signal-regulated kinase (ERK) and c-Jun N-terminal kinase (JNK) phosphorylation remained unchanged (Fig. 4a–d). The p-NF-κB protein levels in the lung tissue of 5 mg/kg PP-instilled mice were significantly higher compared with those in the VC group (Fig. 4a, e).

### Mitochondrial damage and dysfunction in PP-exposed human lung epithelial cells

**Fig. 5:**
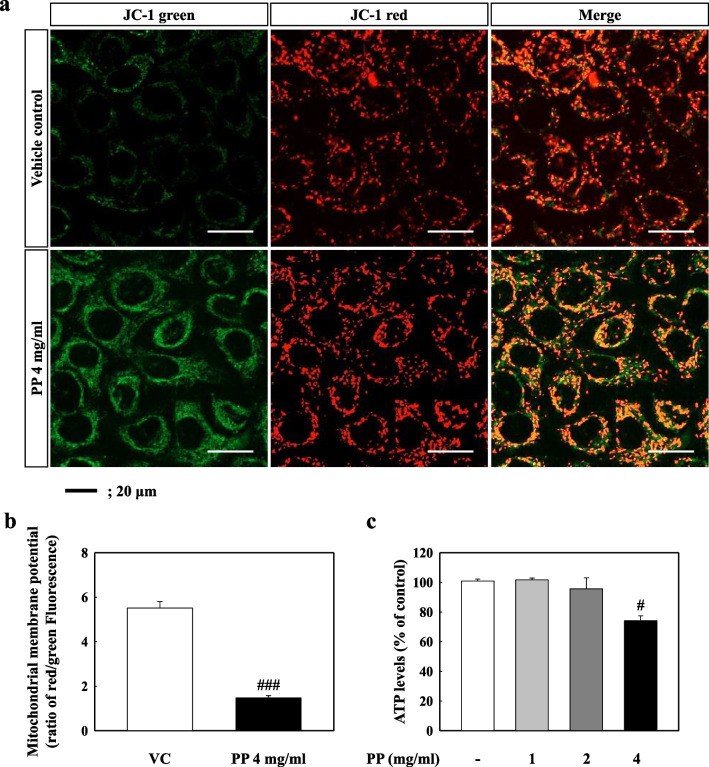
**a** Representative images with 2.5 μg/ml of JC-1 dye for 30 min. Red (568 nm) staining was in J-aggregate form, whereas green (488 nm) staining was in monomer form. **b** Mitochondrial depolarization is indicated by a decrease in the red/green fluorescence intensity ratio. **c** ATP assay was measured in cell lysates of PP-exposed A549 cells. Data are means ± SD (n = 3 per group). ^#^*P* ≤ 0.05; ^###^*P* ≤ 0.001 vs. VC. Scale bar 20 μm

**Fig. 6:**
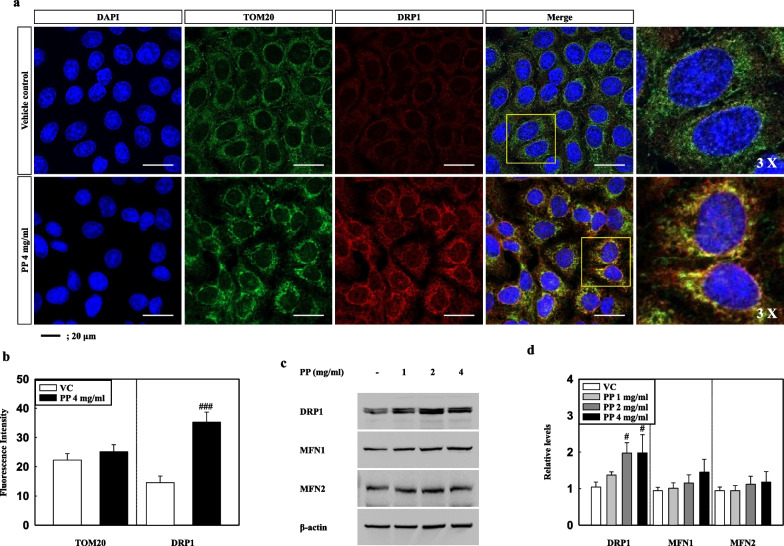
**a** Representative images of TOM20 (green: mitochondrial marker) and DRP1 (red) stained PP-exposed A549 cells. **b** Quantified staining intensities of TOM20 and DRP1. **c** Representative western blotting analysis for DRP1, MFN1, and MFN2 in cell lysates of PP-exposed A549 cells. **d** Relative density analysis of DRP1, MFN1, and MFN2. Data were normalized against β-actin. Data are means ± SD (n = 3 per group). ^#^*P* ≤ 0.05; ^###^*P* ≤ 0.001 vs. VC. Scale bar 20 μm

We investigated the effects of PP stimulation on mitochondrial damage and dysfunction in A549 cells, and found that the mitochondrial membrane potential (ratio of red/green) of 4 mg/mL PP-exposed A549 cells decreased significantly as compared to that of the VC (Fig. 5a, b). In addition, the ATP levels associated with mitochondrial function in 4 mg/mL PP-exposed A549 cells were significantly lower than those of the VC group (Fig. 5c). Confocal microscopy showed strong intracellular fluorescence intensity for DRP1 in the 4 mg/mL PP-exposed A549 cells, however, the same was not observed for TOM20 (Fig. 6a, b). The images showed the merging of the TOM20 and DRP1 proteins in the PP-exposed A549 cells (Fig. 6a). In addition, western blot analysis showed that the protein levels of DRP1 were significantly higher in PP-exposed A549 cells (2 and 4 mg/mL), compared with those in the VC group (Fig. 6c, d). In contrast, the protein levels of MFN1 and MFN2 were slightly increased compared to the control but did not showe significant difference (Fig. 6c, d).

### PP stimulation induces ROS, inflammatory response, and cell deaths in A549 cells

**Fig. 7:**
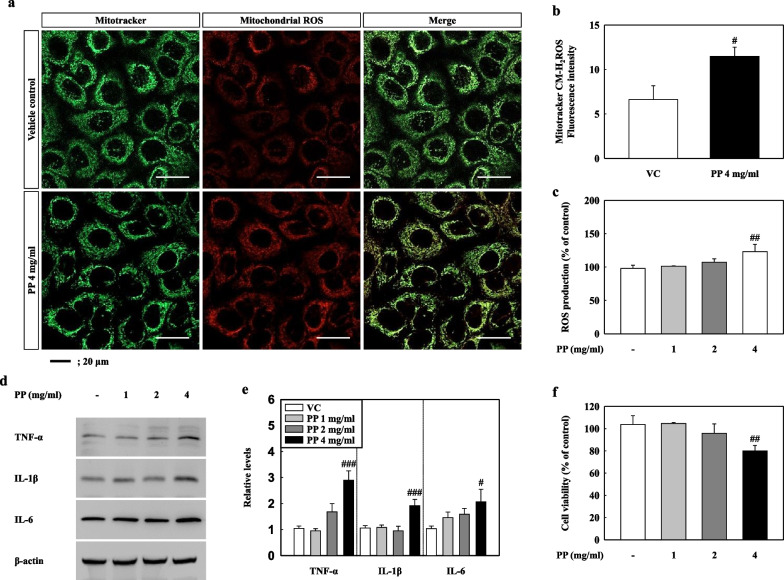
**a** Representative images of mitochondrial (green) and mitochondrial ROS (red) stained PP-exposed A549 cells that were treated with 200 nM for 30 min. **b** Quantified staining intensities for mitochondrial ROS. **c** Total ROS production in PP-exposed A549 cells. **d** Inflammatory cytokine levels of TNF-α, IL-1β, and IL-6 in cell lysate of PP-exposed A549 cells. **e** Relative density analysis of TNF-α, IL-1β, and IL-6. **f** Cell viability assessment by MTT assay. Data were normalized against β-actin. Data are means ± SD (n = 3 per group). ^#^*P* ≤ 0.05; ^##^*P* ≤ 0.01; ^###^*P* ≤ 0.001 vs. VC. Scale bar 20 μm

ROS production, inflammation, and cell death due to mitochondrial damage were measured. ROS production increased significantly in the mitochondria of 4 mg/mL PP-exposed A549 cells compared to the VC (Fig. 7a, b). In addition, the total ROS in the 4 mg/mL PP-treated A549 cells was higher than that of the VC (Fig. 7c). Our results showed that the levels of the inflammatory cytokines TNF-α, IL-1β, and IL-6 increased in the 4 mg/mL PP-exposed A549 cells as compared to the VC (Fig. 7d, e). The cell viability of the 4 mg/mL PP-exposed A549 cells decreased significantly as compared to that of the VC (Fig. 7f). These results indicate that PP stimulation induced ROS production, inflammation, and cell death in A549 cells as a result of mitochondrial damage.

### PP stimulation induces p38-mediated NF-κB nuclear translocation

**Fig. 8:**
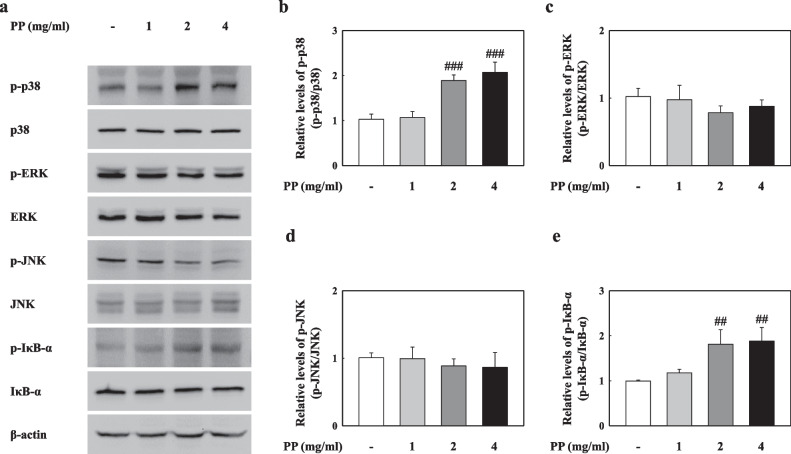
**a** Representative western blotting analysis of p-p38, p38, p-ERK, ERK, p-JNK, JNK, p-IκB-α, and IκB-α in PP-exposed A549 cells. **b** Relative density analysis of p-p38 levels. Data were normalized against p38. **c** Relative density analysis of p-ERK levels. Data were normalized against ERK. **d** Relative density analysis of p-JNK levels. Data were normalized against JNK. **e** Relative density analysis of p-IκB-α levels. Data were normalized against IκB-α. Data are means ± SD (n = 3 per group). ^##^*P* ≤ 0.01; ^###^*P* ≤ 0.001 vs. VC

**Fig. 9:**
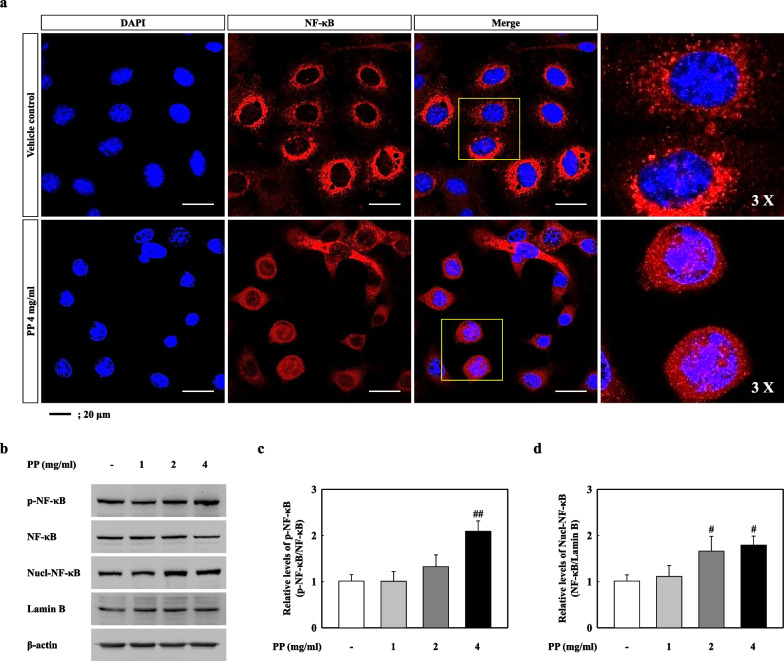
**a** Representative images of DAPI (blue: nuclear) and NF-κB (red) stained PP-exposed A549 cells. **b** Representative western blotting analysis of p-NF-κB, NF-κB, Nuclear-NF-κB, and Lamin B in PP-exposed A549 cells. **c** The relative densities analysis of p-NF-κB levels. Data were normalized against NF-κB. **d** Relative density analysis of Nuclear-NF-κB. Data were normalized against Lamin B. Data are means ± SD (n = 3 per group). ^#^*P* ≤ 0.05; ^##^*P* ≤ 0.01; vs. VC

We observed the MAPK and NF-κB pathways by PP stimulation in vitro. Our results showed that p38 phosphorylation significantly increased in PP-exposed A549 cells, while ERK and JNK remained unchanged (Fig. 8a–d). In addition, IκB-α phosphorylation in the PP-stimulated A549 cells was significantly increased compared to that in VC (Fig. 8a, e). Our results showed that PP stimulation induces the nuclear translocation of NF-κB (Fig. 9). The merging of DAPI and NF-κB proteins was observed in the 4 mg/mL PP-exposed A549 cells (Fig. 9a). PP stimulation significantly increased upon NF-κB phosphorylation compared with that in the VC group (Fig. 9b, c). In addition, the protein levels of NF-κB in the nucleus of PP-stimulated A549 cells significantly increased compared with those in the VC group (Fig. 9b, d).

### p38 and NF-κB activation in PP-exposed A549 cells was regulated by inhibition of ROS

**Fig. 10:**
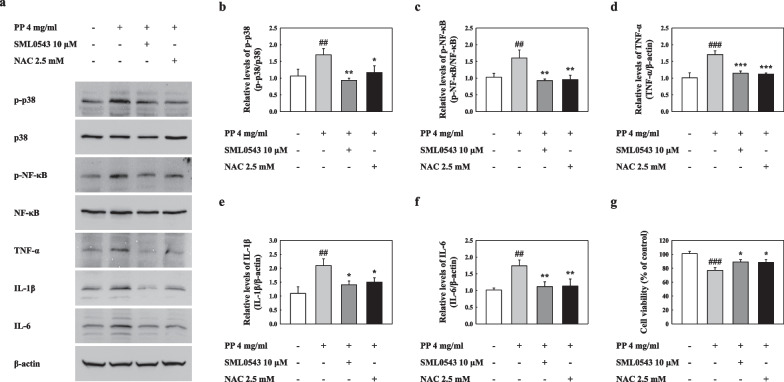
**a** Representative western blotting analysis of p-p38, p38, p-NF-κB, NF-κB, TNF-α, IL-1β, and IL-6 of PP-exposed A549 cells. **b** Relative density analysis of p-p38 levels. Data were normalized against p38. **c** Relative density analysis of p-NF-κB levels. Data were normalized against NF-κB. Relative density analysis of **d** TNF-α, **e** IL-1β, and **f** IL-6. Data were normalized against β-actin. **g** Cell viability assessment by MTT assay. Data are means ± SD (n = 3 per group). ##P ≤ 0.01; ###P ≤ 0.001 vs. VC. *P ≤ 0.05; **P ≤ 0.01; ***P ≤ 0.001 vs. PP

**Fig. 11:**
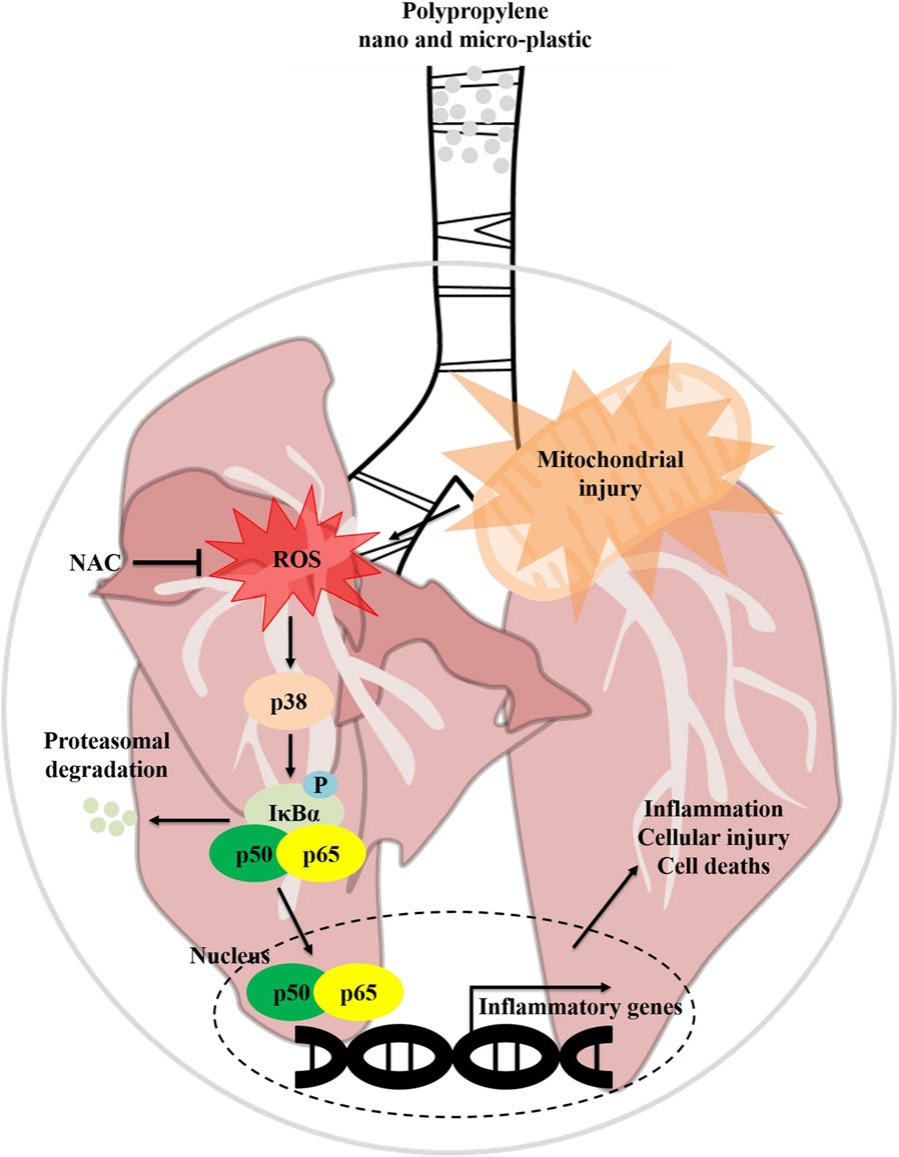
Pathway by which NF-κB signaling is a activated as a result of PP exposure in the lung

Our results showed that the activation of p38 and NF-κB in PP-stimulated A549 cells was significantly decreased by p38 and ROS inhibition (Fig. 10a–c). Inhibiting p38 and ROS in PP-treated A549 cells resulted in a marked reduction in the inflammatory cytokine levels (Fig. 10a, d–f). In addition, the cell viability of PP-exposed A549 cells was recovered by treatment with p38 and ROS inhibitors (Fig. 10g). These results indicate that the ROS production that resulted from PP stimulation contributed to NF-κB activation via p38 phosphorylation (Fig. 11).

## Discussion

The molecular mechanisms of toxicity responses such as inflammation and ROS production to PP nanoplastic stimulation were investigated both in vivo and in vitro. Our results showed increased inflammatory cells, cytokines, and chemokines in the BALF of PP-instilled mice, alongside higher ROS production in the lung tissues, as compared to the VC group. Histopathological analysis of the lung tissue of PP-instilled mice revealed lung damage, including the infiltration of inflammatory cells in the perivascular/parenchymal space, alveolar epithelial hyperplasia, and foamy macrophage aggregates. In vitro investigation revealed depolarisation of the mitochondrial membrane potential, decreased ATP levels, and ROS production as a result of PP stimulation. PP was also found to induce the production of inflammatory cytokines (TNF-α, IL-1β, and IL-6) and cell death, with an increase in the levels of p-p38 and p-NF-κB protein observed both in vivo and in vitro. Interestingly, p38 and ROS inhibition regulated toxic responses such as inflammatory cytokines and cell death. These results suggest that PP nanoplastic stimulation may contribute to the pathogenesis of inflammation within the respiratory system via NF-κB signaling, which is associated with mitochondrial damage (Fig. 11).

With advancements in technology, the exposure to airborne microparticles such as PM in daily life has been increasing. Microparticles including nanoplastic can form as a result of industrial processes, with other sources including traffic or road construction [23–26]. Fragments and fibres are the most commonly found forms of plastic in different environments worldwide [27]. According to a recent report, the human body may be exposed to an average of 0.1–5 g of microplastics per week through different modes such as ingestion and inhalation [28]. Previous studies hypothesised that the major route of human exposure to microplastics was through ingestion. However, recent studies have reported that airborne microplastics through inhalation may be another major route [29, 30]. Airborne microplastic particles have been measured in various regions including indoor and outdoor environments, with particles of approximately 9.80/m^3^ on average, the inhalation rates were predicted to be 15 m^3^/day, with an average rate of annual inhalation exposure of 53,700 particles per person [29, 31]. In a few exposure models, humans were predicted to inhale 6.5–8.97 μg/kg/day of microplastics, and the rate of exposure to microplastics through inhalation, with infants inhaling approximately 3–50-fold higher levels than adults [32]. We observed a pulmonary toxic response including inflammation response and ROS production following 2.5 mg/kg/day of PP nanoplastic exposure, which is shown that correlate to approximately 300-fold the daily human exposure rate for adults and sixfold the dose for infants [32]. These results suggest that further studies on the effects of long-term exposure to microplastics on the lungs are needed, considering that microplastics accumulate in the lungs through continuous and long-term inhalation pathways in the human body.

Inhalation exposure to microplastic in human can be influenced by factors such as the rate of uptake, translocation, and accumulation for the sizes, shapes, doses, and surface functionalization [33]. Microplastics in natural matrices are most often found to have irregular shapes including fragments and fibres and can be deposited in the interior parts of the lung because of their small aerodynamic diameter (particle size expressed in terms of settling rate) [27]. Recent studies reported that microplastics have been identified within all lung regions (upper, middle, and lower) of humans, with the majority being fibrous and fragmented (microplastics with dimensions as small as 4 μm) in shape [10]. Asbestos fibres with lengths ranging from 50 to 200 μm have been found in the alveolar cavity despite their large size, which indicates infiltration into the alveolar region of the lung [34]. Previous studies reported that exposure to airborne microplastics occurring naturally poses might be a risk to the human body through accumulation of lung tissue upon inhalation [7]. However, in vivo studies are lacking on the inhalation toxicity of naturally occurring microplastics with different shapes, including fragments, fibres, and beads. We manufactured PP nanoparticles (irregular in shape and average size 0.66 ± 0.27 μm) by pyrolysis using PP beads. We showed that PP nanoplastics had a spherical shape but formed irregular fragments (Fig. 1a). We observed that inflammatory cellular changes in BALF of PP nanoplastic-instilled mice. Histopathological analysis in 2.5 and 5 mg/kg PP nanoplastic-instilled mice revealed foamy macrophage aggregates in the alveolar space, indicating direct alveolar infiltration of PP nanoplastic. These results show that PP nanoplastic stimulation can cause inflammatory responses through alveolar infiltration; however, further studies are needed to determine the mechanism of lung inflow and injury according to the shape of nanoplastic.

Previous studies have demonstrated that the inhalation of various nanoparticles can lead to bronchial epithelial injury by epithelial barrier infiltration both in vivo and in vitro [22, 35, 36]. Through inhalation, airborne nanoplastics may potentially pose a threat to the health of the human respiratory system. However, only a limited number of in vitro studies have been conducted on the inhalation toxicity of micro and nanoplastic exposure [12]. Previous studies investigated the effects of microplastic exposure for various polymers on lung epithelial cells. It has been reported that Polyvinyl chloride (PVC) microplastic (2.5 mg/mL) stimulation increases the level of inflammatory cytokines in a time-dependent manner. However, exposure to 0.625 mg/mL PVC showed no effect until 48 h [37]. PET and PS nanoplastic exposure for 24 h resulted in ROS production and cytotoxicity owing to the cellular uptake of particles in A549 cells [38, 39]. In other studies, the exposure of A549 cells to PS nanoplastics over 24 h led to the accumulation of the particles, which led to the production of inflammatory cytokines and oxidative stress, with ROS production and lipid peroxidation [40]. However, it has been reported that 4.5 mg/mL PP microplastics (size < 20 μm and 25–200 μm) stimulation did not show any toxicity effect for 48 h [41]. PP has been investigated in several toxicological and physiological studies owing to its use in various disposable products. However, toxicity studies focusing on microplastic inhalation are lacking. Recent reports have suggested that microplastic (1–5 μm in size) exposure can result in infiltration of the lung tissue through the respiratory route, and that nanoplastics can infiltrate the alveolus region [12]. Microplastic (< 10 μm) reduced epithelial uptake by the mucociliary clearance mechanism in the lung, whereas particles (< 1 μm) uptake through the epithelium is possible [6]. Therefore, the inhalation toxicity assessment of PP microplastics should consider nanosized plastics both in vitro and in vivo. Our results showed that ROS production, inflammatory cytokine levels, and cytotoxicity were significantly increased in A549 cells exposed to 4 mg/mL of PP nanoplastic compared to those in control cells (Fig. 7), and alveolar epithelial hyperplasia and inflammatory cell infiltration were observed in PP nanoplastic-instilled mice (2.5 and 5 mg/kg) (Fig. 3). In contrast, the cytotoxicity in 1 and 2 mg/mL PP nanoplastic stimulation were slightly increased compared to the VC, but did not show significant increase. These results suggest that PP nanoplastics from long-term exposure and dose might contribute to the pulmonary toxic response through lung epithelium injury.

Our results showed that the number of inflammatory cells, including macrophages, neutrophils, and lymphocytes in the BALF of PP nanoplastic-instilled mice (2.5 and 5 mg/kg) increased significantly as compared with that in the VC group. Neutrophils, which are the most common leukocytes and are essential first responders during the initial phases of inflammation, were predominant in the BALF of PP-instilled mice (Fig. 2a). The granulation and activation of neutrophils causes pulmonary inflammation via the release of various inflammatory cytokines and chemokines [42–44]. Especially, helper T (Th) cytokines play important roles in inflammatory responses in pulmonary diseases such as asthma and pulmonary fibrosis [45, 46]. Previous studies reported that IL-17 overexpression and neutrophil accumulation in BALF of mice with diesel exhaust particulates (DEP)-induced lung inflammation were observed and that the Th17 pathway might be involved in DEP-induced inflammation [23]. We investigated the gene expression patterns after PP exposure to reveal the molecular mechanism associated with PP-induced lung inflammation. We observed that the expressions of Th17 signaling pathway-associated genes including those encoding C–C motif chemokine ligand (CCL) 2, CCL12, CCL17, CXCL1, and CXCL5 were increased in the lung tissue of PP-instilled mice. We presume that, PP stimulation might have induced lung inflammation through the Th17 signaling pathway (Additional file 2: Table S1). Recently, nanoparticles such as TiO2, CeO2, and ZnO have been reported to activate neutrophil degranulation, inducing inflammatory tissue injury [47–49]. PM-instilled mice have previously been shown to suffer from an increase in the number of neutrophils followed by the release of inflammatory cytokines and chemokines [50]. Interestingly, our results showed that inflammatory cytokines and chemokines such as TNF-α, IL-1β, IL-6, MCP-1, and CXCL1/KC in the BALF of PP-instilled mice (2.5 or 5 mg/kg) increased significantly as compared with those in the VC group, and the histopathological results of PP-instilled mice showed inflammatory cell infiltration (Figs. 2b–f, 3). These results indicate that PP nanoplastic stimulation may contribute to neutrophilic lung inflammation.

Oxidative stress and endoplasmic reticulum (ER) stress resulting from cellular organelle injury caused by exposure to environmental factors such as chemicals and pathogens can lead to various diseases including pulmonary diseases via abnormal inflammation and immune responses [19, 20, 51, 52]. Our results show that PP nanoplastic-exposed A549 cells had significantly increased ROS production and levels of antioxidant enzymes including catalase (CAT), superoxide dismutase (SOD)1, SOD2, and glutathione peroxidase-1 (GPX1) (Additional file 1: Figure S1). However, the protein levels of ER stress markers such as binding immunoglobulin protein (BiP) and C/EBP homologous protein (CHOP) were unchanged (Additional file 1: Figure S2). Interestingly, nuclear factor erythroid-2-related factor 2 (Nrf2) protein levels (total and nuclear) in PP-exposed A549 cells were significantly increased compared to the control, which might have regulated the expression of antioxidant proteins that protect against oxidative damage (Additional file 1: Figure S3). Although more studies focusing on the mechanism of mitochondrial damage by uptake, translocation, and accumulation for PP nanoplastic exposure, are required, we speculate that oxidative stress may be an indirect result of mitochondrial damage. Previous studies have reported that nanoparticles can damage mitochondria, and induce toxicity [53–56]. Recent studies have reported that ultrafine dust exposure induced oxidative stress and mitochondrial damage in bronchial epithelial cells (BEAS-2B) and monocyte/macrophage cell lines (RAW 264.7 cells) [53]. NH_2_-PS stimulation induces mitochondrial dysfunction, leading to decreased ATP levels, DNA degradation, and a decline in the mitochondrial membrane potential of BEAS-2B and RAW 264.7 cells [54]. Our results showed that PP nanoplastic exposure induces mitochondrial dysfunctions such as mitochondrial depolarization and decreases ATP levels (Fig. 5). Interestingly, DRP1 proteins merged into the damaged mitochondrial regions (Fig. 6), which might be a quality control mechanism for preserving a healthy mitochondrial network via fission [57]. These results suggest that PP nanoplastic stimulation causes mitochondrial damage and that long-term PP nanoplastic exposure may potentially lead to mitochondrial diseases.

Recent studies have reported that airborne microplastics including nanoplastic cause inflammation through various pathogenesis, such as dust overload, oxidative stress, and cytotoxicity [58–61]. In particular, ROS overproduction by particle exposure-induced inflammation and cytotoxicity is mediated by the release of cytokines and inflammatory mediators due to the translocation of nuclear factor NF-κB in cell signaling pathways [62–64]. Various studies have reported that oxidative stress causes NF-κB activation via the phosphorylation of MAPKs such as p38, ERK, and JNK, which regulate important cellular processes such as proliferation, stress responses, apoptosis, and immune defence [65–69]. Recent studies have reported that the persistent activation of p38 significantly contributes to the pathogenesis of Th2 low neutrophilic inflammation, which is associated with severe asthmatic phenotypes [70]. In asthmatic patients, activated p38 MAPK contributed to TNF-α secretion from natural killer (NK) cells stimulated by IL-12, and the secretion of IL-6, IL-8, and MCP-1 were also partially dependent upon p38 activation [71, 72]. Interestingly, recent studies have reported that apoptosis was induced through p38 signaling by hydrogen peroxide, which is used as an oxidative stress inducer [73]. We demonstrated that PP nanoplastic stimulation caused inflammatory response, oxidative stress, and cell death. Interestingly, we also observed that the inflammatory cytokines and cell death induced by PP nanoplastic stimulation were reduced by p38 and ROS inhibitors (Fig. 11). These results suggest that PP nanoplastic stimulation may contribute to pulmonary toxic response via the p38 MAPK and NF-κB signalling pathways.

## Conclusions

The toxicological effects that PP nanoplastics have on the respiratory system were investigated, with results indicating that PP stimulation leads to lung inflammation in vivo. PP exposure causes mitochondrial injury and cytotoxicity manifested as ROS production, inflammatory responses, and cell death in vitro. Inflammation and cell death of PP-exposed A549 cells were reversed by p38 and ROS inhibition. These results suggest that PP nanoplastics contribute to the pathogenesis of inflammation via p38-mediated NF-κB signaling, resulting from mitochondrial injury in the respiratory systems.

## Methods

### PP nanoplastics

PP particles were prepared by precipitating a solution dissolved at 200 °C on a hot plate with PP beads (Sigma, 428116) and xylene (DAEJUNG, 8587–4410) solvent with ethanol. PP dispersal solutions were prepared by bath sonication for 30 min after addition of 1% DMSO in saline. FE-SEM (JSM-7100F) analysis was performed to measure the shape and size of the PP particles, and dynamic light scattering (DLS; ELSZ-2000) was used to measure the sizes of the PP particles with regards to their dispersal in the liquid. Dispersion stability was measured using Turbiscan (Turbiscan LAB) in both in vivo and in vitro experiments.

### Animals and experimental design

Seven-week-old male ICR mice weighing 35.57 ± 1.21 g were purchased from Orient Bio Inc. (Seongnam, Korea). The mice were housed in a temperature-controlled environment (22 ± 3 °C) with a relative humidity of 50 ± 20%, a 12 h light/dark cycle, and ventilated with air (10–20 times/h). The mice were provided with pellets that are specifically produced for experimental animals (PMI Nutrition International, Richmond, IN, USA) and UV-irradiated (Steritron SX-1; Daeyoung, Seoul, Korea) and filtered (1 μm-pore filter) tap water. All experimental procedures were approved by the Institutional Animal Care and Use Committee at the Korea Institute of Toxicology (IACUC #2108-0003). The mice in the PP groups received intratracheal instillation of 1, 2.5, or 5 mg/kg PP in 50 μl saline solution five times per week for 4 weeks using an automatic video instillator [74]. The mice in the VC group were instilled with saline using the same method. The body weights of the mice were measured on days 1, 2, 4, 8, 11, 15, 18, 22, 26, and 29 (Additional file 1: Figure S4). The mice were sacrificed on day 29.

### Cell culture and treatment

A549 cells were obtained from the American Type Culture Collection (ATCC). Cells were grown in RPMI 1640 medium (Gibco) containing 10% inactivated foetal bovine serum (FBS; Gibco). Cells were seeded at a concentration of 2 × 10^5^ in 0.5 mL of medium using sterile 6-well culture plates. The A549 cells were incubated at 37 °C in a humidified 5% CO2 atmosphere and treated with PP (1, 2, and 4 mg/mL) for 16 h. The mitochondrial ROS inhibitor N-acetyl-L-cysteine (NAC; Sigma, A7250) was added 6 h before PP treatment. A p38 MAP kinase inhibitor (Sigma, SML0543) was added 2 h before PP treatment (Additional file 3).

### BALF preparation

At 24 h after the last PP intratracheal instillation, the mice were anesthetised with isoflurane and exsanguinated. The left lung was ligated, and the right lung was gently lavaged three times via the tracheal tube with a total volume of 0.7 mL phosphate-buffered saline (PBS; Gibco). The collected solutions were pooled and maintained at 4 ℃. The total cells in the BALF were counted with a NucleoCounter (NC-250; ChemoMetec, Gydevang, Denmark). To differentiate the cell types, BAL cells were prepared using Cytospin (Thermo Fisher Scientific) and stained with Diff-Quik solution (Dade Diagnostics, Aguada, Puerto, USA). A total of 200 cells were counted on each slide.

### Measurement of inflammatory cytokine and chemokine levels in BALF

The TNF-α, IL-1β, IL-6, MCP-1, and KC levels in BALF were quantified by ELISA using a commercial kit (R&D Systems) in accordance with the manufacturer’s protocols.

### Histopathological analysis

The left lung of each mice was fixed with 10% neutral-buffered formalin (NRF). The specimens were dehydrated and embedded in paraffin to produce tissue blocks which were sectioned into 4-µm thick slices. Lung sections from each animal were stained with haematoxylin and eosin (H&E). All samples were analysed using a Leica DM2500 microscope (Leica Instruments, Wetzlar, Germany) at 200× and 400× magnifications. The degree of lung injury in each animal was scored on a scale as follow: 0, no symptoms; 1, minimal; 2, slight; 3, moderate; 4, severe.

### Cell viability and ROS measurement

Cell viability was evaluated using an MTT assay (Sigma, M2128). To determine cell viability, a 1 mg/mL MTT solution was added to the cells after PP stimulation before incubation at 37 °C for 3 h [75]. Absorbance was measured using Synergy Mx microplate reader (BioTek) at OD 570 nm. ROS levels in the lung tissue of PP-instilled mice were quantified using commercial ELISA kits (Abbkine, KTE71621) in accordance with the manufacturer’s protocols. The cells were then incubated with 3% serum in PBS containing 1 μM CM-H2DCFDA dye for 30 min at 37 °C and the number of stained cells determined using a flow cytometer (Beckman Coulter). Mitochondrial ROS levels were measured using MitoTracker Red CM-H2XRos (Invitrogen, M7513) and MitoTracker Green FM (Invitrogen, M7514). Staining was incubated with 3% serum in PBS containing 200 nM of Mitotracker Red CM-H2XRos and MitoTracker Green FM for 30 min at 37 °C. Stained cells were analysed using a Zeiss lsm 800 confocal microscope (Carl Zeiss) at 400× magnification.

### Preparation of cell lysates and western blot analysis

Cell lysates were homogenised in the presence of a protease inhibitor cocktail with RIPA buffer (Thermo Fisher Scientific). Nuclear extracts were homogenised using a nuclear and cytoplasmic extraction kit (Thermo Fisher Scientific, 78835) according to the instructions of the manufacturer. Protein concentrations were determined using Bradford reagent (Bio-Rad). The samples were then loaded onto an SDS-PAGE gel. After electrophoresis at 90 V for 120 min, the protein was transferred to polyvinylidene difluoride membranes (Merck Millipore) at 250 mA over 60 min using a transfer method. Nonspecific sites were blocked with 5% non-fat dry milk in Tris-buffered saline/Tween 20 (TBS-T) for 1 h and incubated with DRP1 (NOVUS, NB110-55288), MFN1 (Proteintech, 13798-1-AP), MFN2 (Proteintech, 12186-1-AP), Lamin B (Cell signaling, 9087S), p-p38 (Cell signaling, 4511S), p38 (Cell signaling, 8690S), p-ERK (Cell signaling, 4370S), ERK (Cell signaling, 4695S), p-JNK (Cell signaling, 4668T), JNK (Cell signaling, 9252S), p-IκB-α (Cell signaling, 9246S), IκB-α (Cell signaling, 4812S), p-NF-κB (Cell signaling, 3033S), NF-κB (Cell signaling, 8242S), IL-1β (Abcam, ab9722), IL-6 (Invitrogen, P620), TNF-α (Abcam, ab66579), CAT (Abcam, ab16731), SOD1 (Abcam, ab16831), SOD2 (R&D Systems, MAB3419), GPX1 (Abcam, ab108427), BiP (Cell signaling, 3183S), CHOP (Cell signaling, 2895S), Nrf2 (Cell signaling, 12721S), and β-actin (Santa Cruz, sc-47778) overnight at 4 ℃. Anti-rabbit (Cell signaling, 7074S) and anti-mouse (Cell signaling, 7076S) horseradish peroxidase-conjugated IgG was used to detect antibody binding. The binding of specific antibodies was visualized using the iBright CL 1000 imaging system (Thermo Fisher Scientific) after treatment with the ECL reagent (Thermo Fisher Scientific). The results of the densitometric analysis were expressed as the relative ratio of the target protein to the reference protein. The relative ratio of the target protein to the control was arbitrarily denoted 1. The original data for western blot results (Additional file 3).

### Measurement of mitochondrial membrane potential

Mitochondrial membrane potential was measured using JC-1 dye (Invitrogen, T3168). Cells were incubated with 3% serum in PBS containing 2.5 μg/mL of JC-1 for 30 min at 37 °C. Stained cells were analysed using a Zeiss lsm 800 confocal microscopes (Carl Zeiss) at 400× magnification. The cells were scanned by dual excitation with 488 nm (green) and 568 nm (red) laser lines.

### Immunofluorescence staining and confocal microscopy

Cells were fixed in methanol at − 20 °C for 3 min. After washing with PBS, the cells were blocked with 1% BSA in PBS-T for 30 min. Cells were then washed three times with PBS and incubated overnight at 4 °C with TOM20 (Santa Cruz, sc-17764), DRP1 (NOVUS, NB110-55288), and NF-κB (Cell signaling, 8242S). The sections were treated with Alexa Fluor 488 anti-mouse (Invitrogen, A11001) and Alexa Fluor 594 anti-rabbit (Invitrogen, A11037) for 2 h at RT. Stained cells were analysed using a Zeiss lsm 800 confocal microscopes (Carl Zeiss) at 400× magnification.

### Analysis of ATP levels

ATP levels were assessed using a colorimetric ATP assay kit (Abcam, ab83355), according to the instructions of the manufacturer. ATP levels were measured using a Synergy Mx microplate reader (BioTek) at 570 nm.

### Statistical analysis

Statistical analysis was performed using GraphPad InStat v. 3.0 (GraphPad Software, Inc., La Jolla, CA, USA). Statistical comparisons were performed using one-way ANOVA followed by Dunnett’s multiple comparison test, and statistical comparisons between two groups were performed using the Student’s t-test. Data are presented as the arithmetic mean ± SD. A value of *p* < 0.05 was considered to indicate statistically significant results.

## Supplementary Information

**Additional file 1.** **Fig. S1**. (a) Representative western blotting analysis of CAT, SOD1, SOD2, and GPX1 of PP-exposed A549 cells. (b) Relative density analysis of CAT, SOD1, SOD2, and GPX1 levels. Data were normalized against β-actin. Data presented as are mean ± SD (n = 3 per group). ^#^*P* ≤ 0.05; ^##^*P* ≤ 0.01; ^###^*P* ≤ 0.001 vs. VC. **Fig. S2**. (a) Representative western blotting analysis of BiP and CHOP of PP-exposed A549 cells. (b) Relative density analysis of BiP levels. (c) Rela tive density analysis of CHOP levels. Data were normalized against β-actin. Data presented as are mean ± SD (n = 3 per group). **Fig. S3**. (a) Representative western blotting analysis of Nrf2 (Total and Nuclear) of PP-exposed A549 cells. (b) Relative density analysis of Nrf2 (Total and Nuclear) levels. Data were normalized against β-actin and Lamin B. Data presented as are mean ± SD (n = 3 per group). ^##^*P* ≤ 0.01 vs. VC. **Fig. S4**. (a) Effect of PP on changes in body weights of mice. The body weights of mice were measured on Days 1, 2, 4, 8, 11, 15, 18, 22, 26, and 29. Data presented as are mean ± SD (n = 6 per group).

**Additional file 2.** Supplementary methods and supplementary Table 1. **Table S1**. Genes altered in the Th17 signaling pathway.

**Additional file 3.** The original data for western blot results.

## Data Availability

The datasets used and/or analyzed during the current study are available from the corresponding author on reasonable request.

## Abbreviations

ATP: Adenosine triphosphate
BiP: Binding immunoglobulin protein
BALF: Bronchoalveolar lavage fluid
CAT: Catalase
CCL: C-C motif chemokine ligand
CHOP: C/EBP homologous protein
COPD: Chronic obstructive pulmonary disease
CXCL1/KC: C-X-C motif chemokine ligand 1
DEP: Diesel exhaust particulates
DRP1: Dynamin-related protein 1
ER: Endoplasmic reticulum
ERK: Extracellular signal-regulated kinases
GPX1: Glutathione peroxidase-1
JNK: C-Jun N-terminal kinases
IL: Interleukin
MFN1: Mitofusin 1
MFN2: Mitofusin 2
MAPK: Mitogen-activated protein kinase
MCP-1: Monocyte chemoattractant protein-1
NK: Natural killer
Nrf2: Nuclear factor erythroid-2-related factor 2
NF-κB: Nuclear factor kappa B
PM: Particulate matter
PE: Polyethylene
PET: Polyethylene terephthalate
PP: Polypropylene
PS: Polystyrene
ROS: Reactive oxygen species
SOD: Superoxide dismutase
Th: Helper T
TNF-α: Tumor necrosis factor-α
VC: Vehicle control
