# Supplementary figures and images for: Polypropylene nanoplastic exposure leads to lung inflammation through p38-mediated NF-κB pathway due to mitochondrial damage
Source: Part Fibre Toxicol. 2023 Jan 10;20:2. doi: 10.1186/s12989-022-00512-8 (PMC9829531; doi:10.1186/s12989-022-00512-8)

a

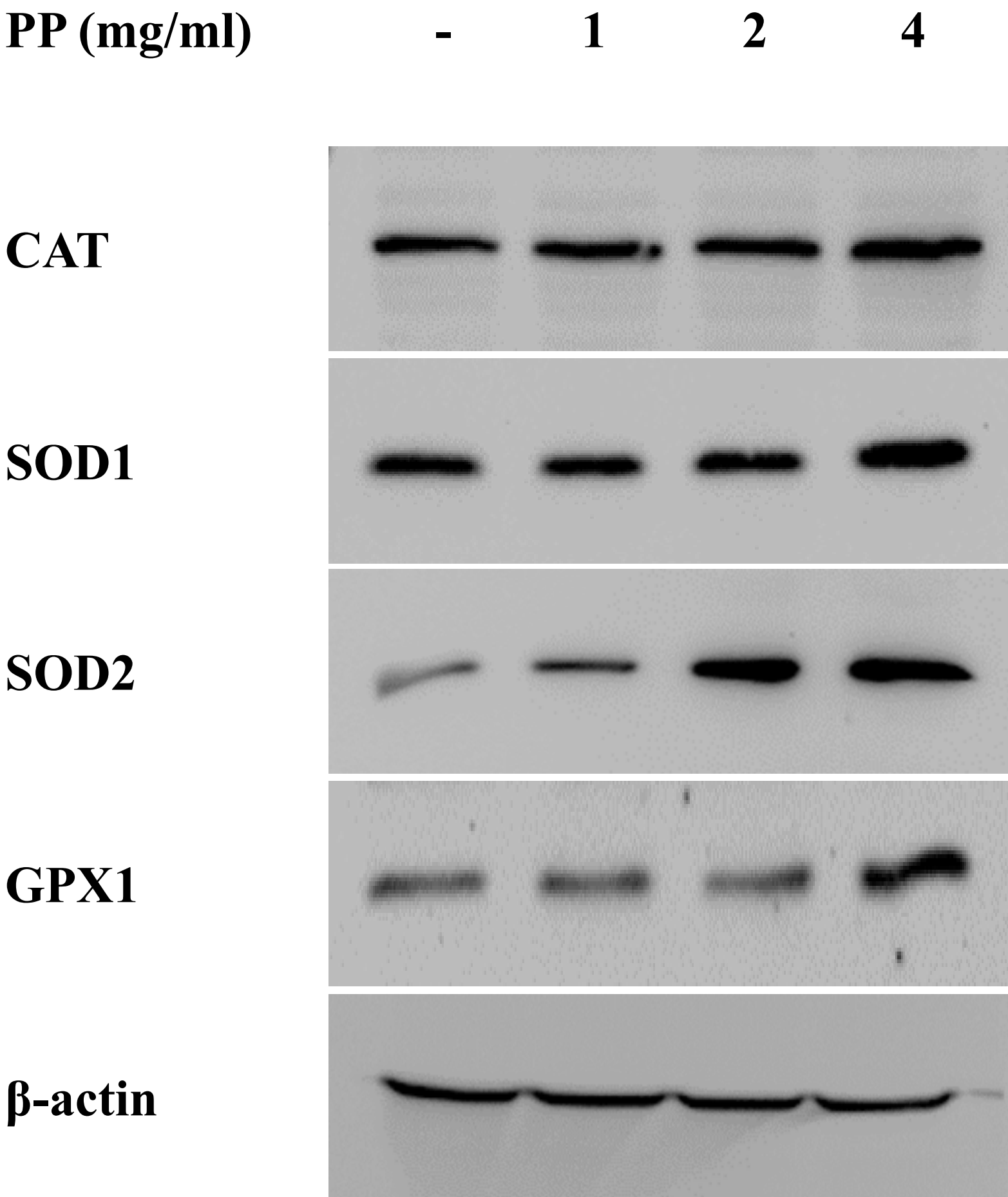

b

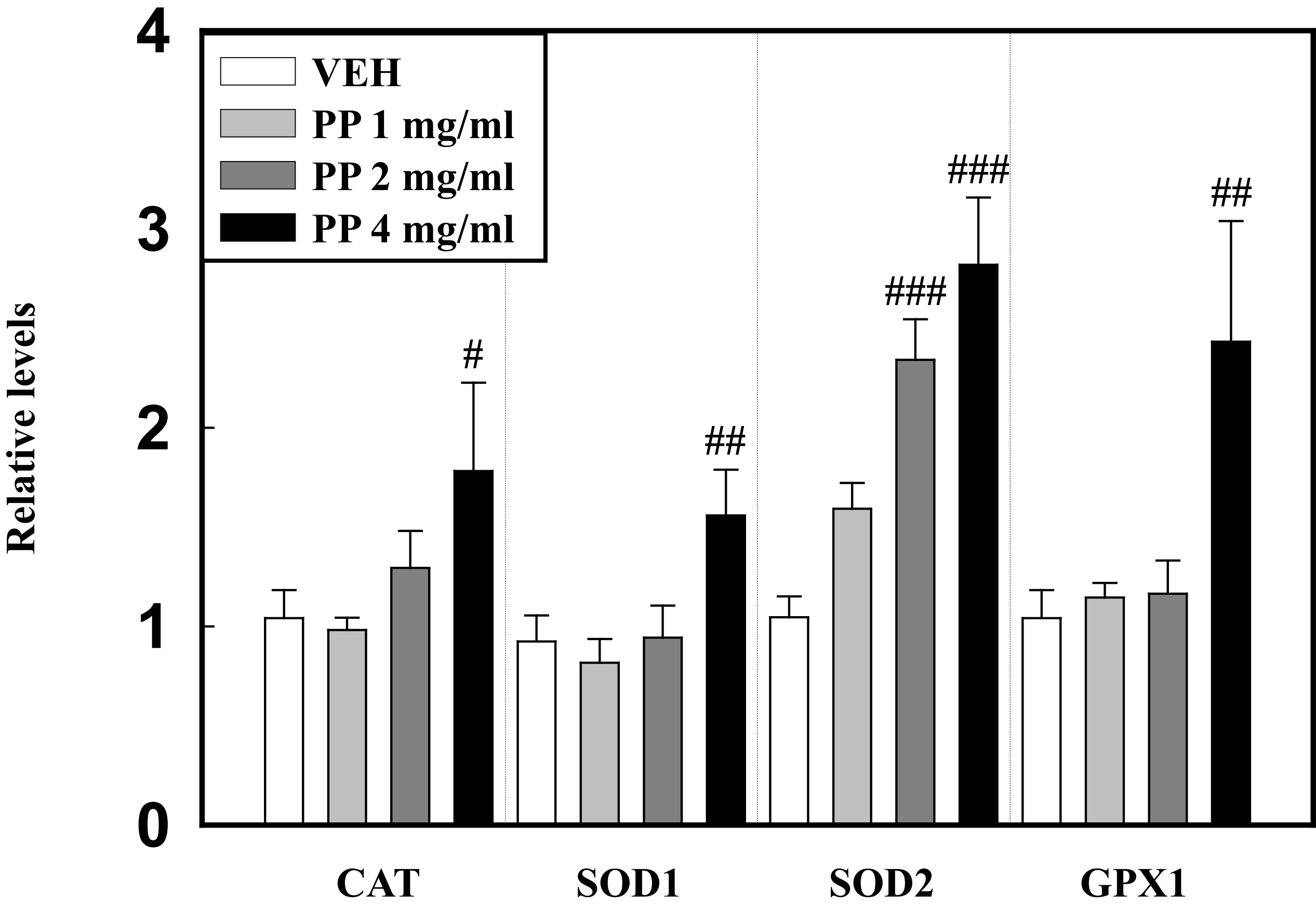

**a**

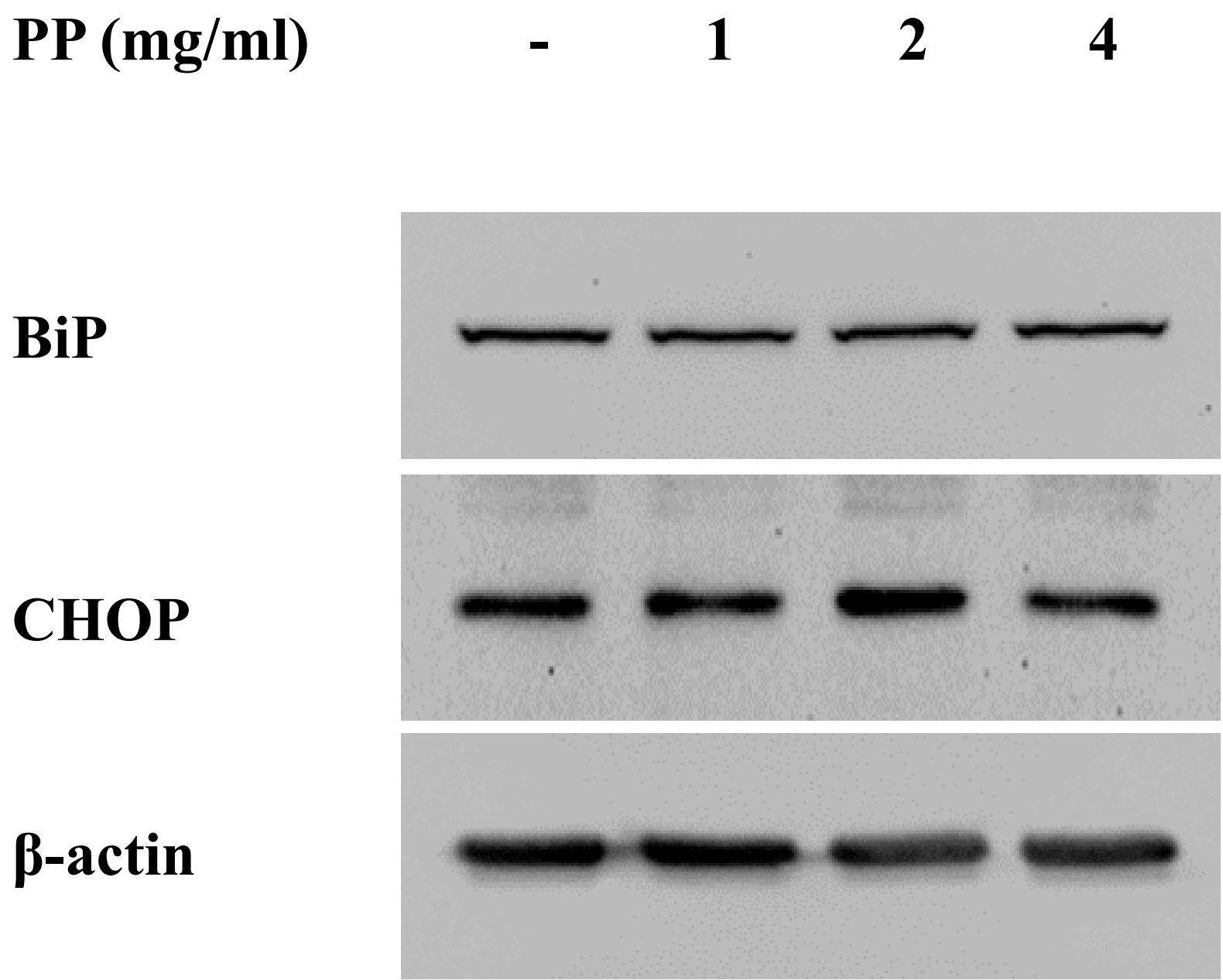

**b**

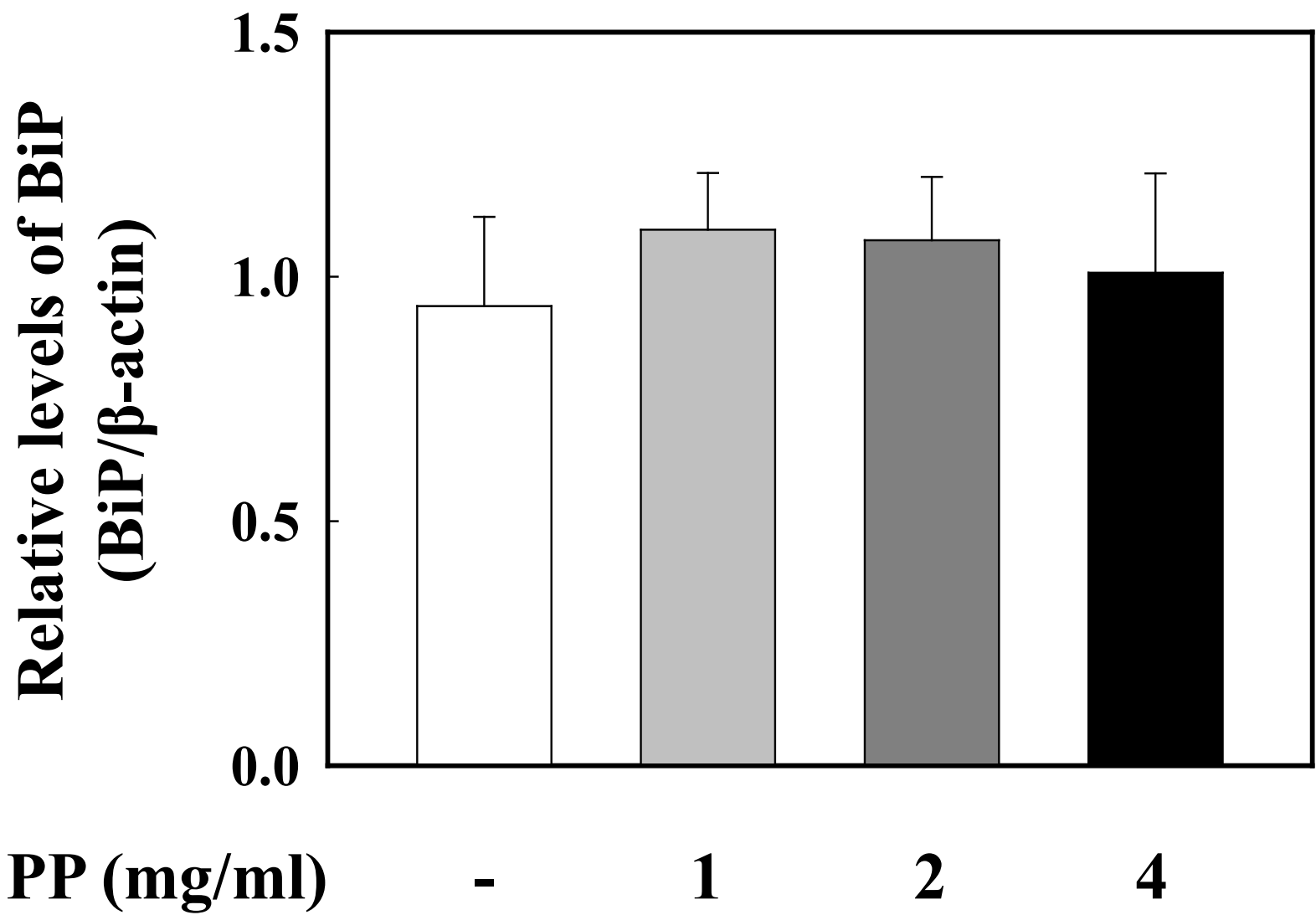

**c**

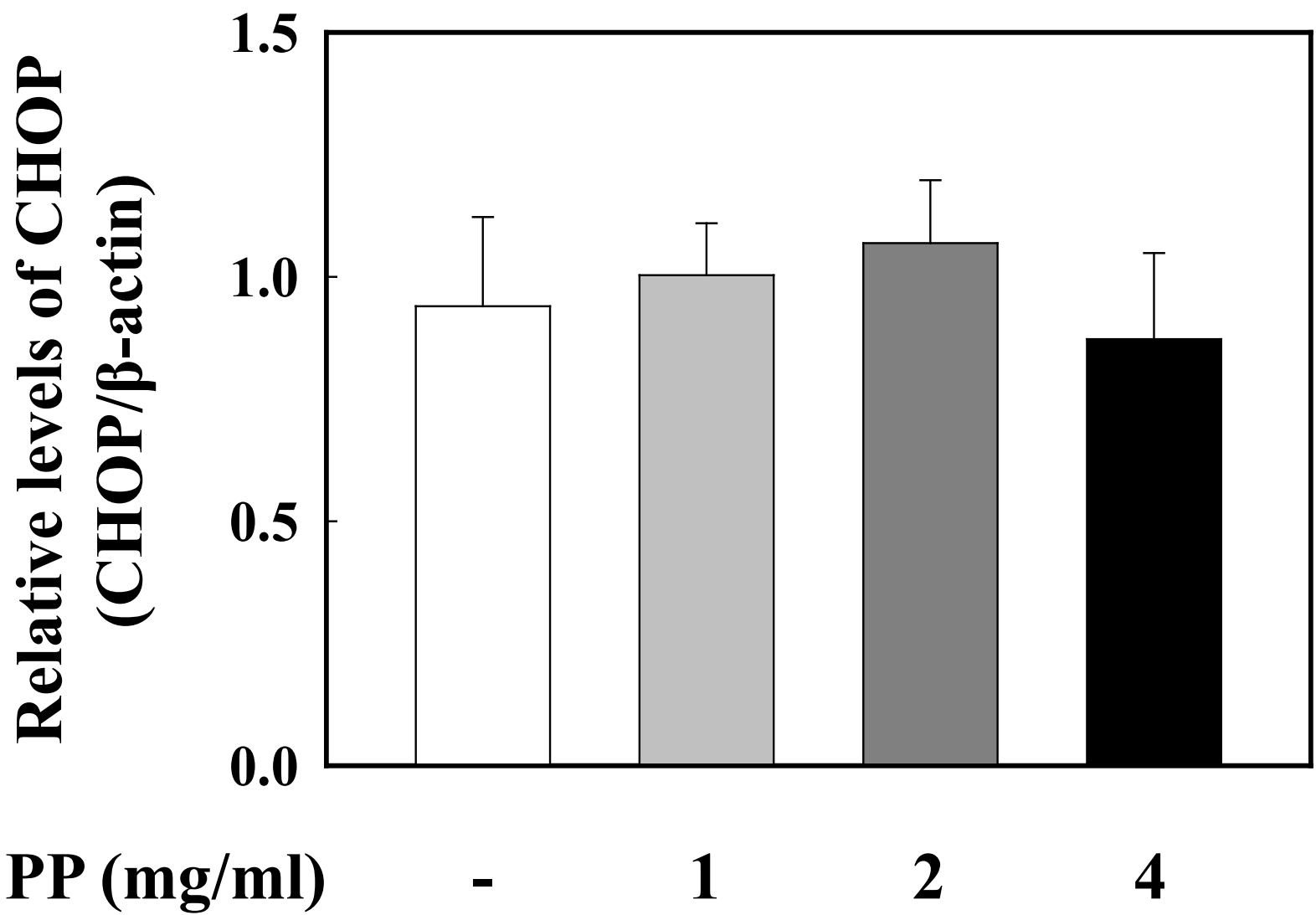

a

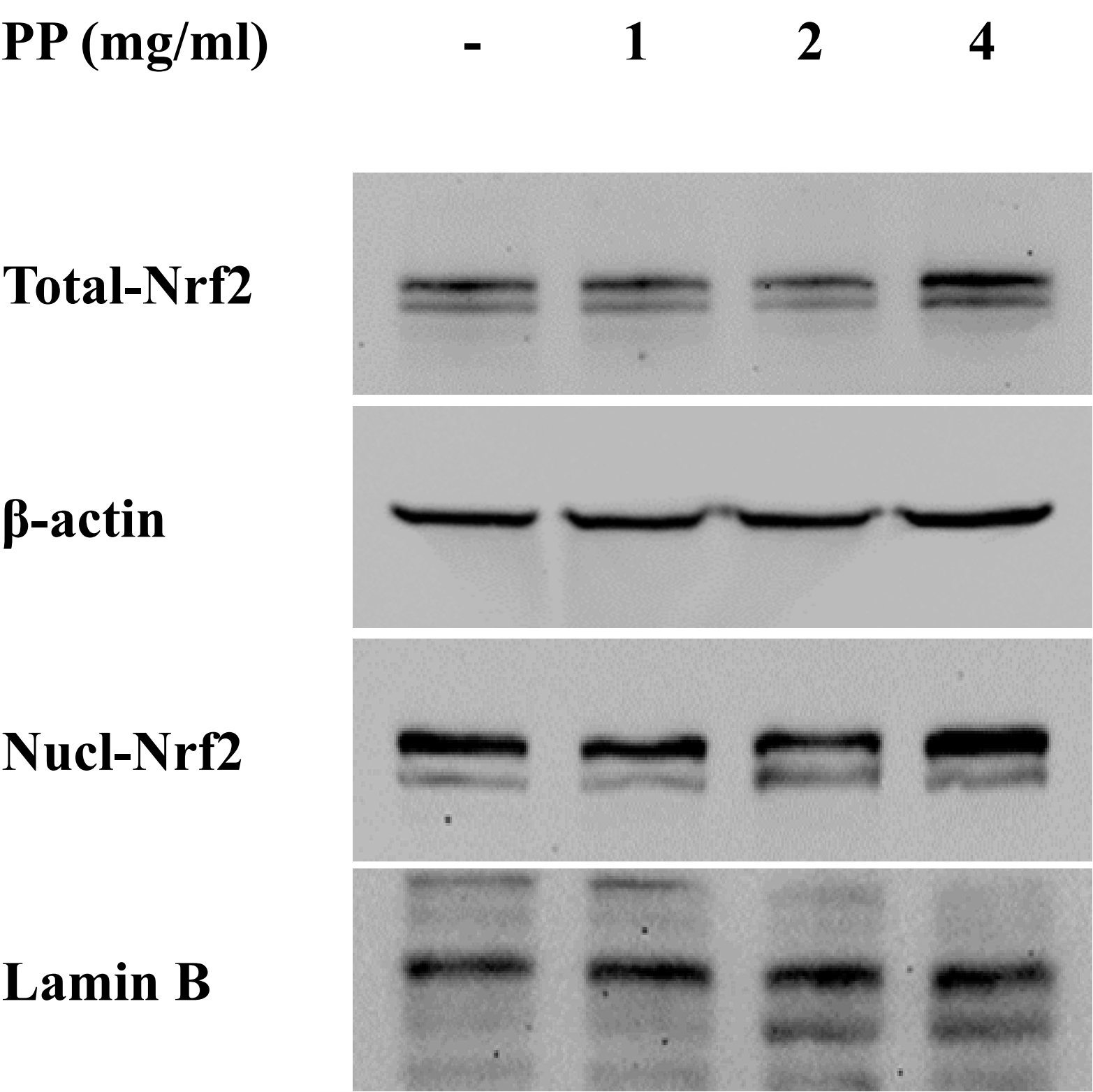

b

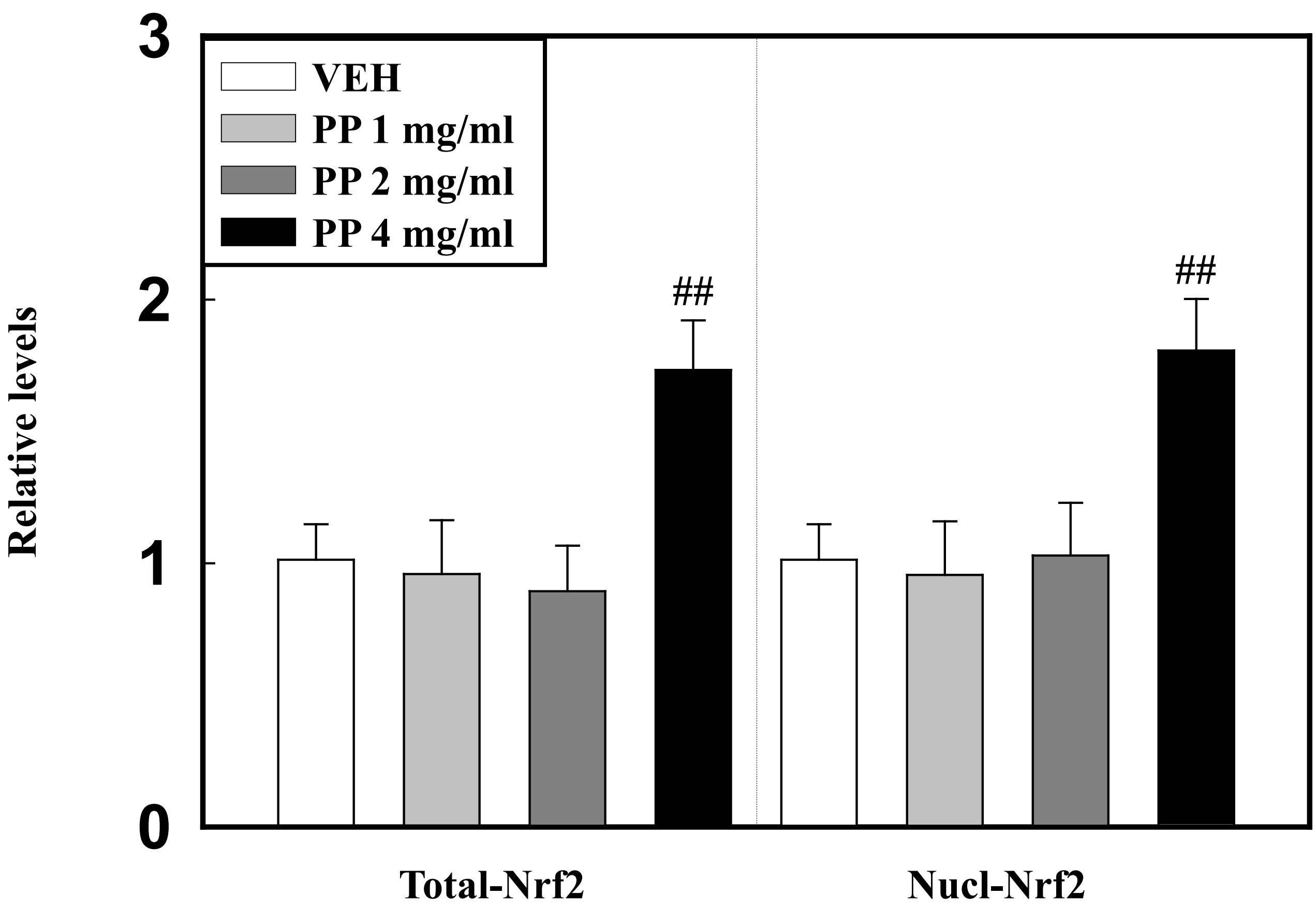

a

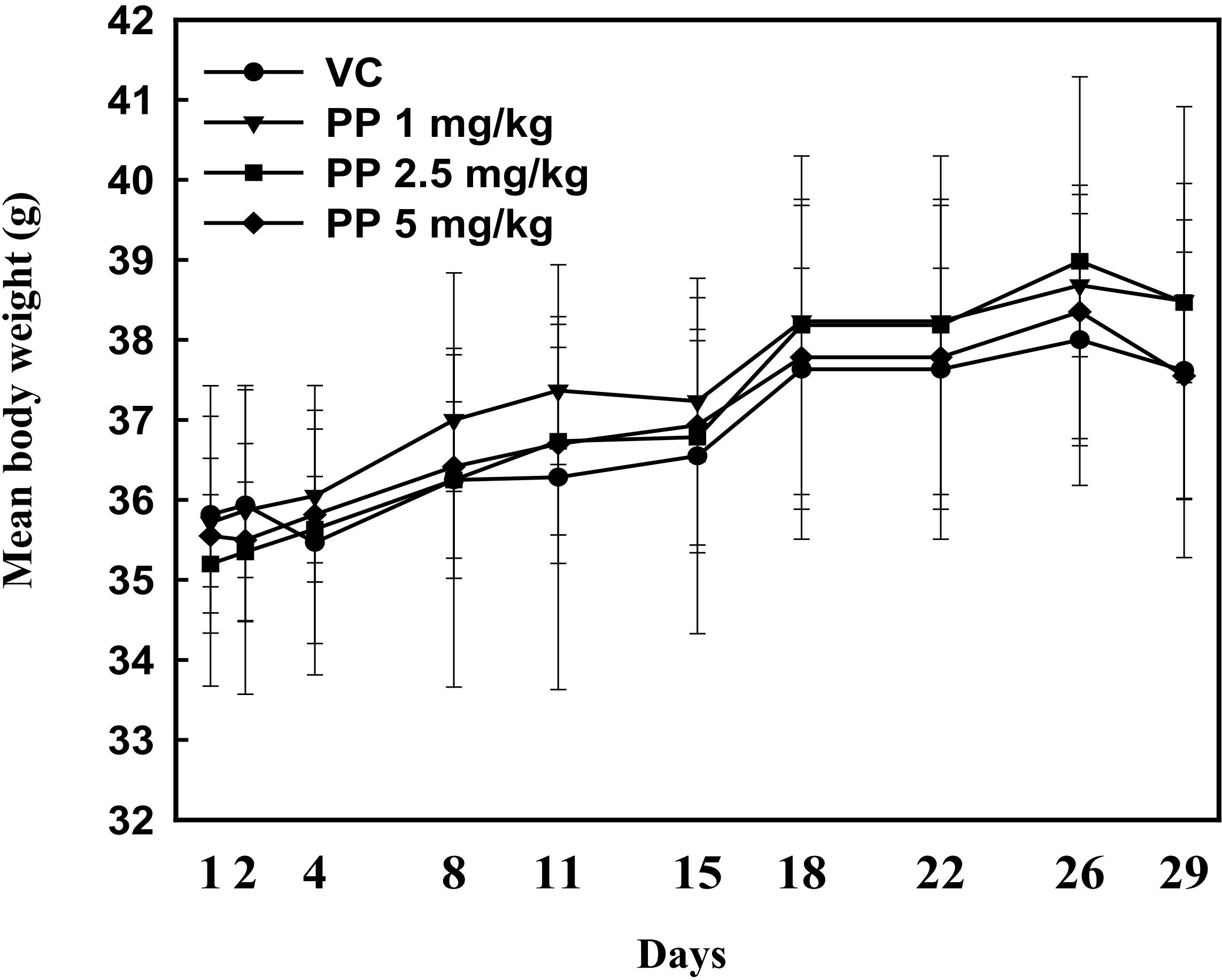

Supplement: Supplementary file 1 — Additional file 1. Fig. S1. (a) Representative western blotting analysis of CAT, SOD1, SOD2, and GPX1 of PP-exposed A549 cells. (b) Relative density analysis of CAT, SOD1, SOD2, and GPX1 levels. Data were normalized against β-actin. Data presented as are mean ± SD (n = 3 per group). #P ≤ 0.05; ##P ≤ 0.01; ###P ≤ 0.001 vs. VC. Fig. S2. (a) Representative western blotting analysis of BiP and CHOP of PP-exposed A549 cells. (b) Relative density analysis of BiP levels. (c) Rela tive density analysis of CHOP levels. Data were normalized against β-actin. Data presented as are mean ± SD (n = 3 per group). Fig. S3. (a) Representative western blotting analysis of Nrf2 (Total and Nuclear) of PP-exposed A549 cells. (b) Relative density analysis of Nrf2 (Total and Nuclear) levels. Data were normalized against β-actin and Lamin B. Data presented as are mean ± SD (n = 3 per group). ##P ≤ 0.01 vs. VC. Fig. S4. (a) Effect of PP on changes in body weights of mice. The body weights of mice were measured on Days 1, 2, 4, 8, 11, 15, 18, 22, 26, and 29. Data presented as are mean ± SD (n = 6 per group). [file 12989_2022_512_MOESM1_ESM.pdf]
