# Supplementary material for: Polypropylene nanoplastic exposure leads to lung inflammation through p38-mediated NF-κB pathway due to mitochondrial damage
Source: Part Fibre Toxicol. 2023 Jan 10;20:2. doi: 10.1186/s12989-022-00512-8 (PMC9829531; doi:10.1186/s12989-022-00512-8)
Supplement: Supplementary file 2 — Additional file 2. Supplementary methods and supplementary Table 1. Table S1. Genes altered in the Th17 signaling pathway. [file 12989_2022_512_MOESM2_ESM.docx]

**Supplementary Methods**

**1.1 RNA isolation, library preparation, and sequencing**

Total RNA was isolated using Trizol reagent (Invitrogen). RNA quality was assessed using Agilent 2100 bioanalyzer with the RNA 6000 Nano Chip (Agilent Technologies, Amstelveen, Netherlands), and RNA quantification was performed using a ND-2000 Spectrophotometer (Thermo Fisher Scientific). Only samples with an A260/A280 ratio >1.8 and RIN value >7 were considered suitable for use. For control and test RNAs, a library was constructed using QuantSeq 3′ mRNA-Seq Library Prep Kit (Lexogen, Inc., Austria) according to the manufacturer’s instructions. Each 500-ng sample of total RNA was prepared and an oligo-dT primer containing an Illumina-compatible sequence at its 5′ end was hybridized, and reverse transcription performed. After degradation of the RNA template, second-strand synthesis was initiated by a random primer containing an Illuminacompatible linker sequence at its 5′ end. The double-stranded library was purified by using magnetic beads to remove all reaction components. The library was amplified to add the complete adapter sequences required for cluster generation. The finished library was purified from PCR components. High-throughput sequencing was performed through single-end 75 sequencing using NextSeq 500 (Illumina, Inc., USA).

**1.2 Data analysis**

QuantSeq 3′ mRNA-Seq reads were aligned using Bowtie2. Bowtie2 indices were either generated from genome assembly sequences or the representative transcript sequences for aligning to the genome or transcriptome. The alignment file was used for assembling transcripts, estimating their abundances, and detecting differential expression of genes. Differentially expressed genes (DEGs) were determined based on counts from unique and multiple alignments using coverage in Bedtools (Quinlan AR, 2010). Read count (RC) data were processed based on the quantile normalization method using EdgeR within R and Bioconductor. Gene classification was based on searches in the DAVID (http://david.abcc.ncifcrf.gov/) and Medline databases (http://www. ncbi.nlm.nih.gov/).

**1.3 Gene ontology (GO) category and pathway analysis**

To classify the genes altered by PP exposure into groups with a similar pattern of expression, each gene was assigned to an appropriate category according to its main cellular function. To determine significantly over-represented GO findings, the DAVID functional annotation clustering tool was used by choosing the default option. Pathway analysis was performed to determine significant pathways for DEGs using microarray gene pathway annotations downloaded from KEGG (http://www.genome.jp/kegg/). A Fisher exact test was used to identify significantly enriched pathways and the resulting P values were adjusted using the BH false discovery rate (FDR) algorithm. Pathway categories with FDR < 0.05 were reported. Additional pathways-gene associations were analyzed using the Comparative Toxicogenomics Database (CTD, http://ctdbase.org).

**Figure Legends**

**Supplementary 1.** (a) Representative western blotting analysis of CAT, SOD1, SOD2, and GPX1 of PP-exposed A549 cells. (b) Relative density analysis of CAT, SOD1, SOD2, and GPX1 levels. Data were normalized against β-actin. Data presented as are mean ± SD (n = 3 per group). ^#^P ≤ 0.05; ^##^P ≤ 0.01; ^###^P ≤ 0.001 vs. VC.

**Supplementary 2.** (a) Representative western blotting analysis of BiP and CHOP of PP-exposed A549 cells. (b) Relative density analysis of BiP levels. (c) Relative density analysis of CHOP levels. Data were normalized against β-actin. Data presented as are mean ± SD (n = 3 per group).

**Supplementary 3.** (a) Representative western blotting analysis of Nrf2 (Total and Nuclear) of PP-exposed A549 cells. (b) Relative density analysis of Nrf2 (Total and Nuclear) levels. Data were normalized against β-actin and Lamin B. Data presented as are mean ± SD (n = 3 per group). ^##^P ≤ 0.01 vs. VC.

**Supplementary 4.** (a) Effect of PP on changes in body weights of mice. The body weights of mice were measured on Days 1, 2, 4, 8, 11, 15, 18, 22, 26, and 29. Data presented as are mean ± SD (n = 6 per group).

**Supplementary** **Table 1. Genes altered in the Th17 signaling pathway**

| Th17 Activation Pathway |  | | |
| --- | --- | --- | --- |
| Symbol | Entrez Gene Name | Fold change |  |
| CCL2 | chemokine (C-C motif) ligand 2 | 2.823 |  |
| CCL12 | chemokine (C-C motif) ligand 12 | 1.876 |  |
| CCL17 | chemokine (C-C motif) ligand 17 | 3.013 |  |
| CXCL1 | chemokine (C-X-C motif) ligand 1 | 3.242 |  |
| CXCL5 | chemokine (C-X-C motif) ligand 5 | 16.929 |  |
