# Supplementary material for: Polypropylene nanoplastic exposure leads to lung inflammation through p38-mediated NF-κB pathway due to mitochondrial damage
Source: Part Fibre Toxicol. 2023 Jan 10;20:2. doi: 10.1186/s12989-022-00512-8 (PMC9829531; doi:10.1186/s12989-022-00512-8)

Figure 4.

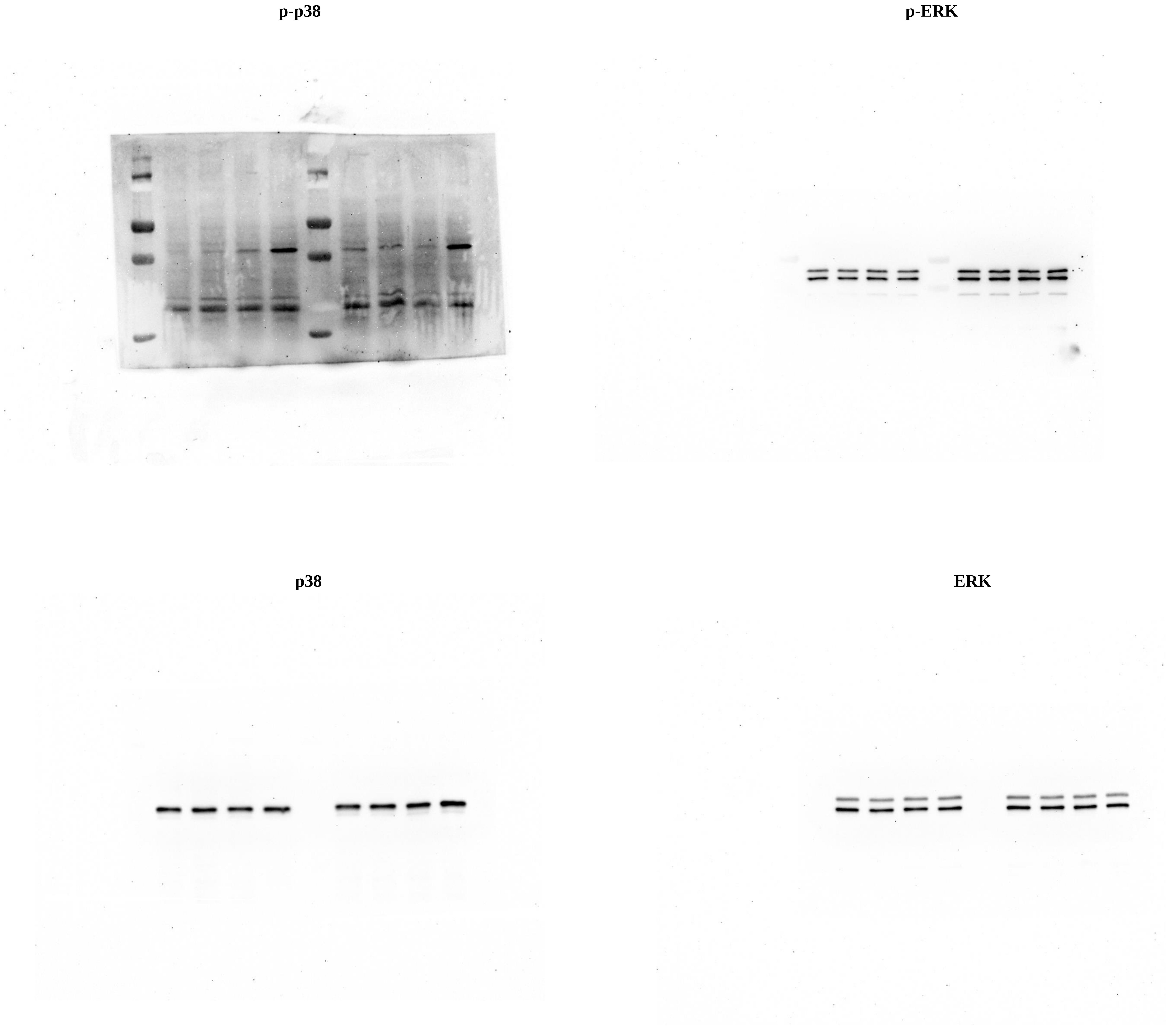

Figure 4.

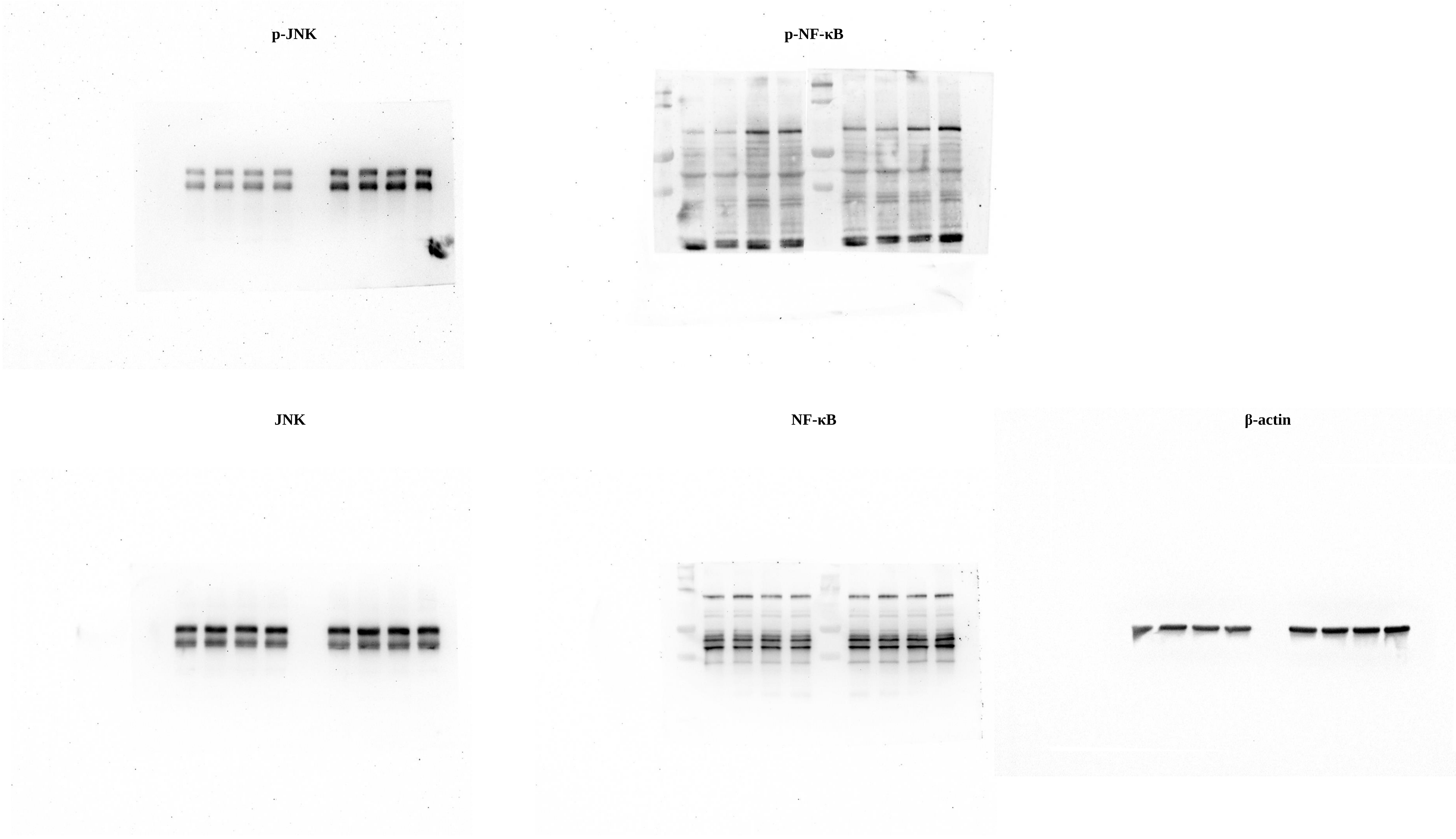

Figure 6.

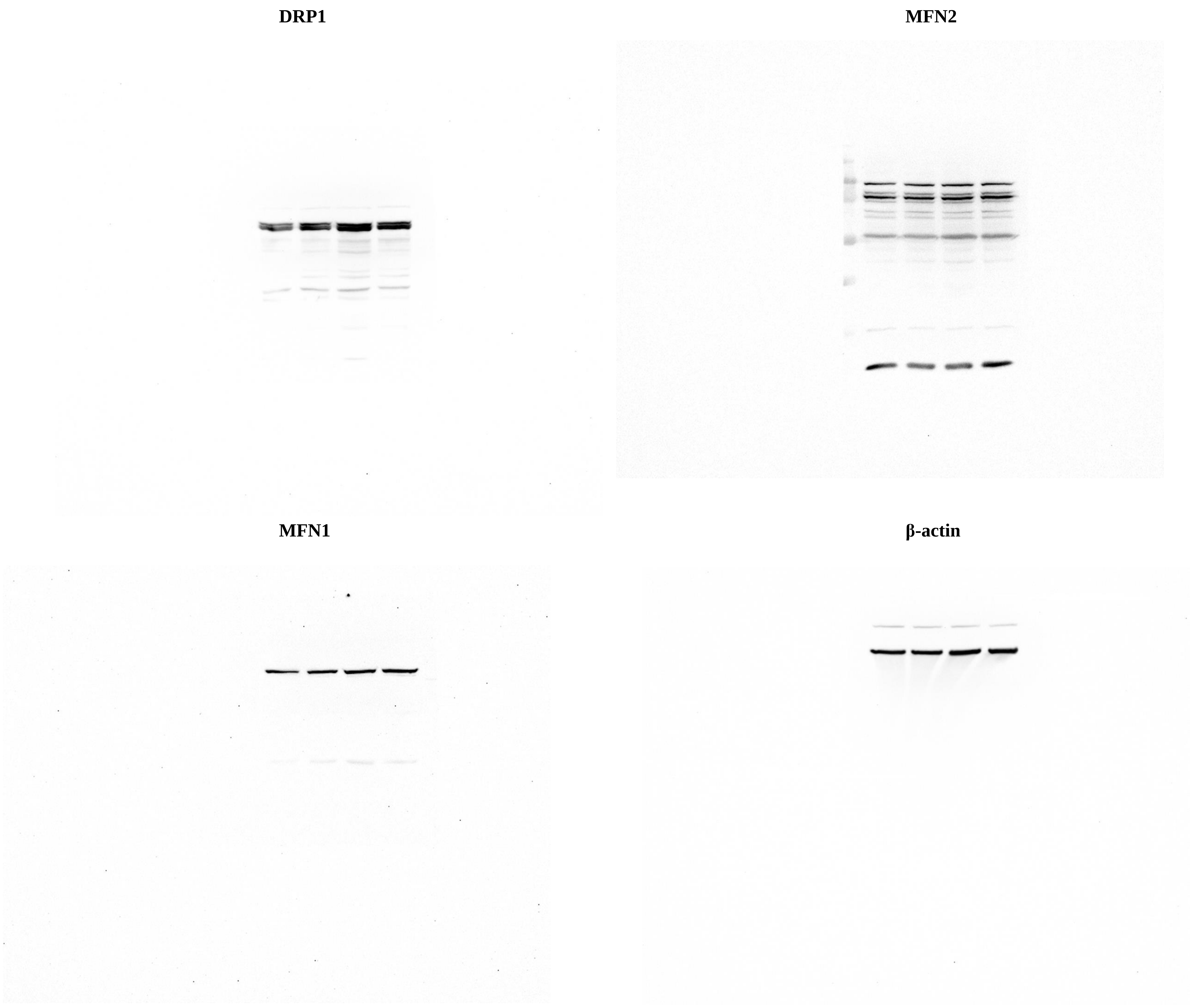

Figure 7.

TNF-α

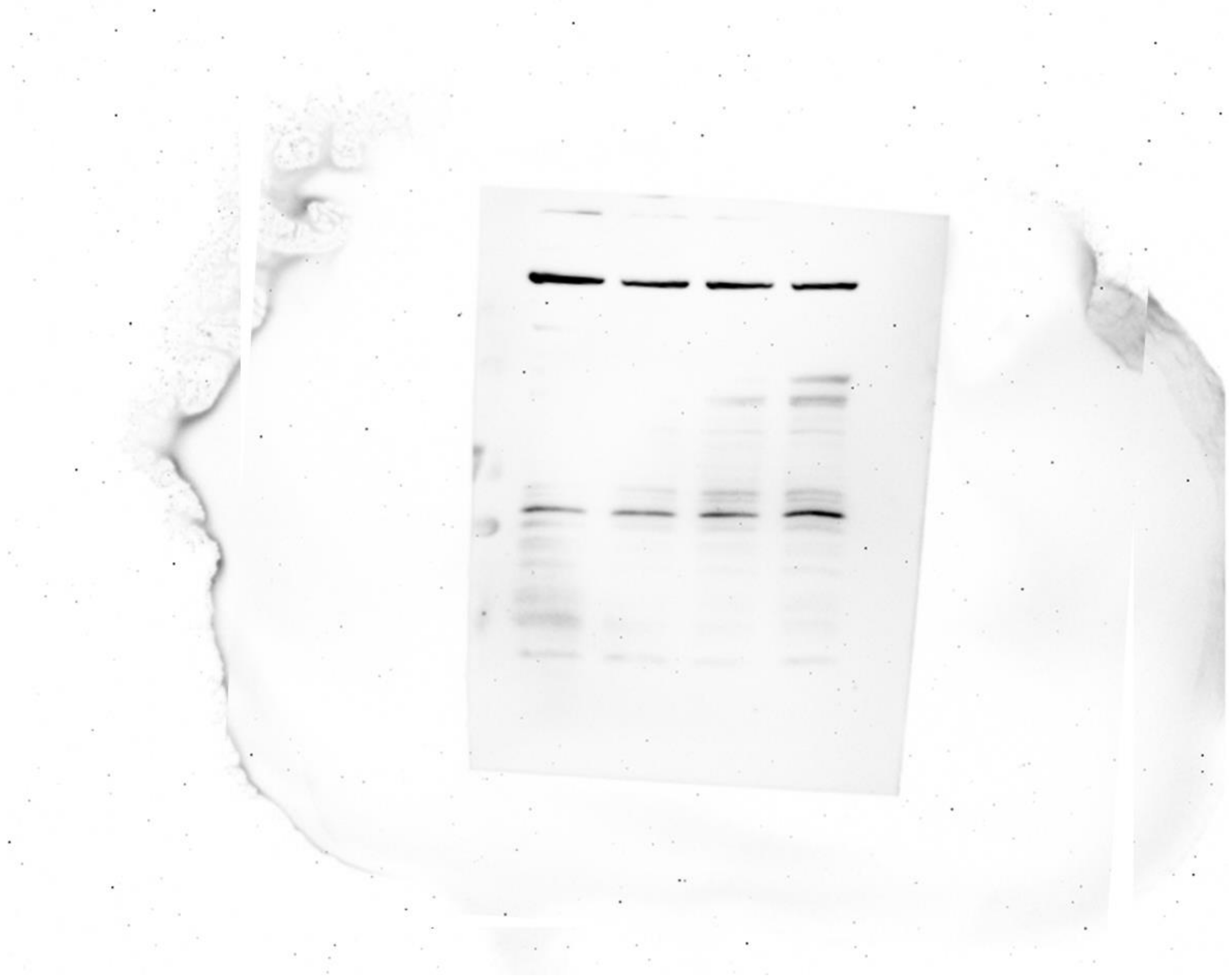

IL-6

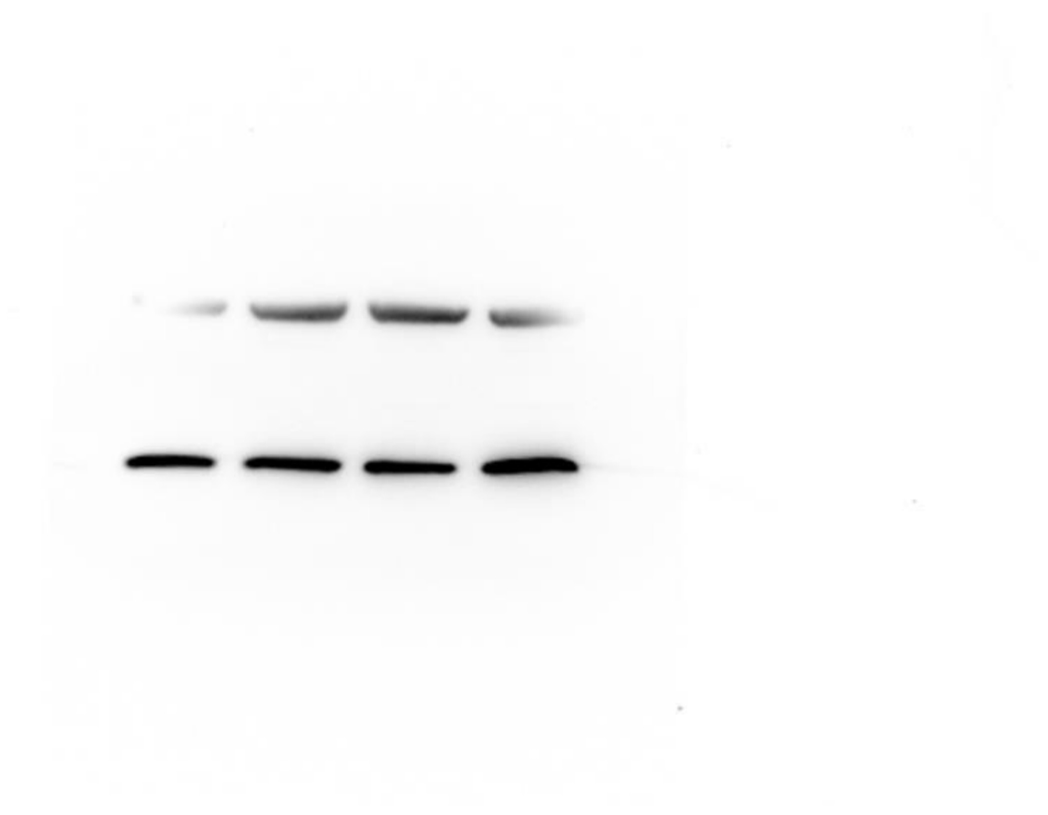

β-actin

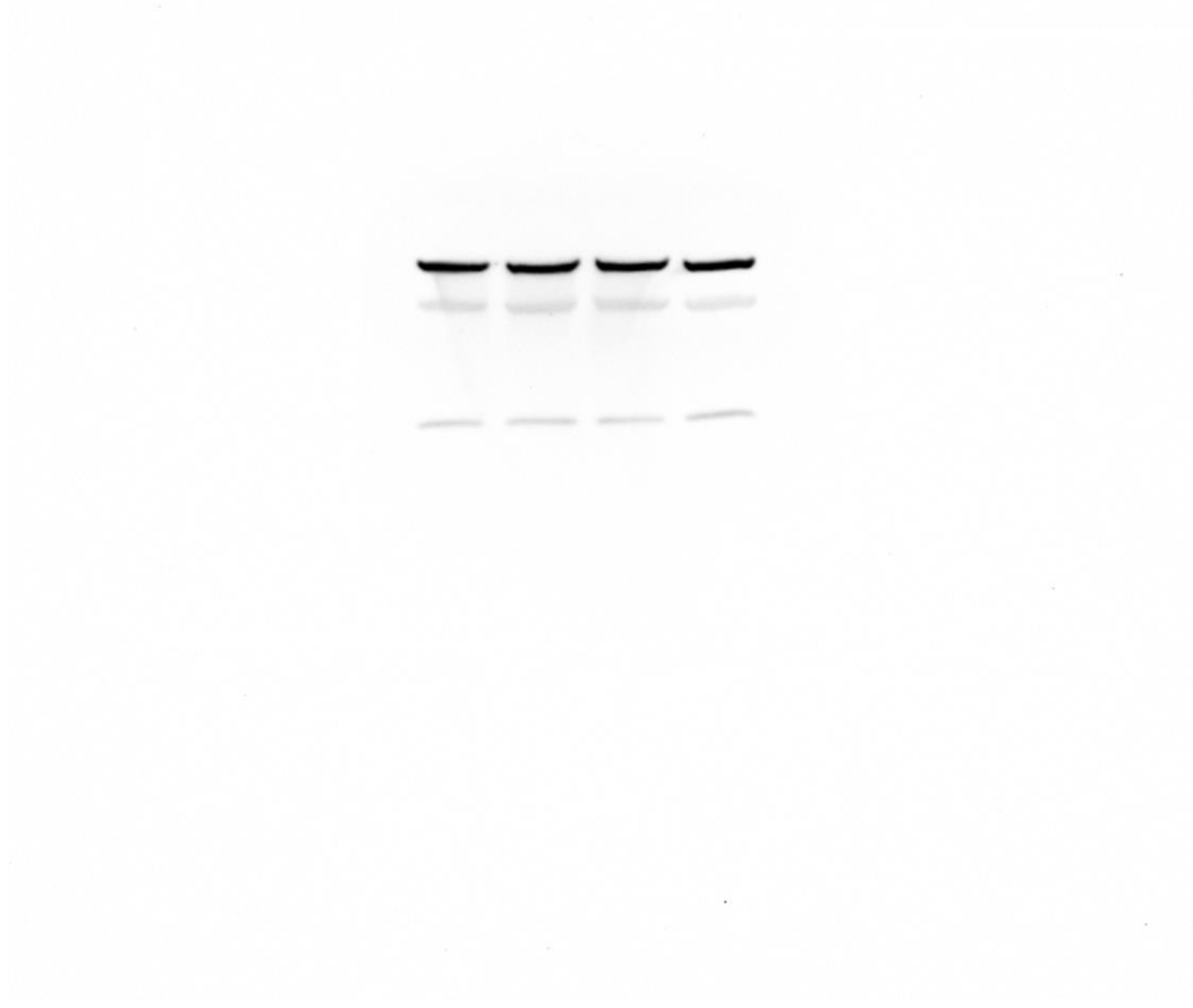

IL-1β

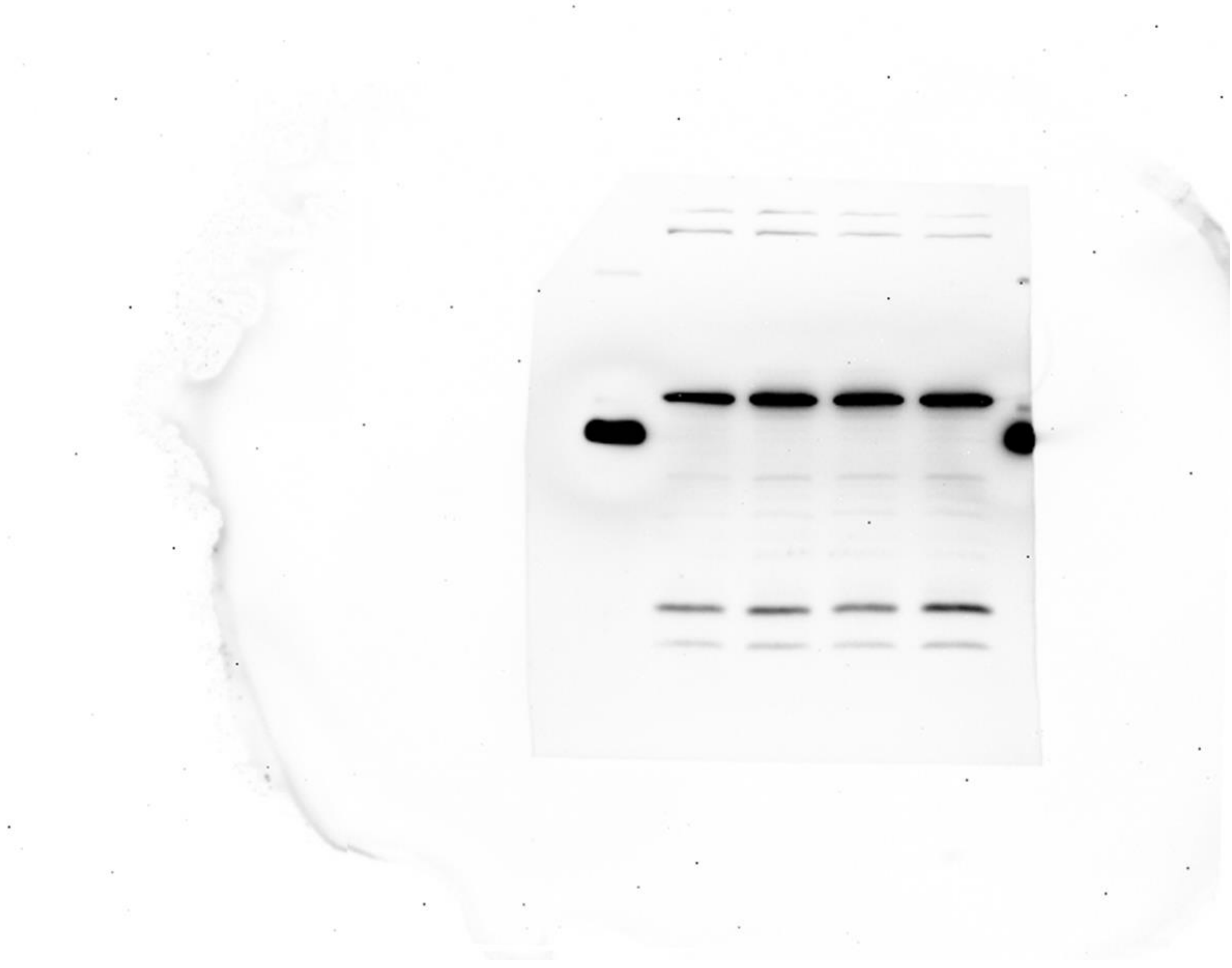

Figure 8.

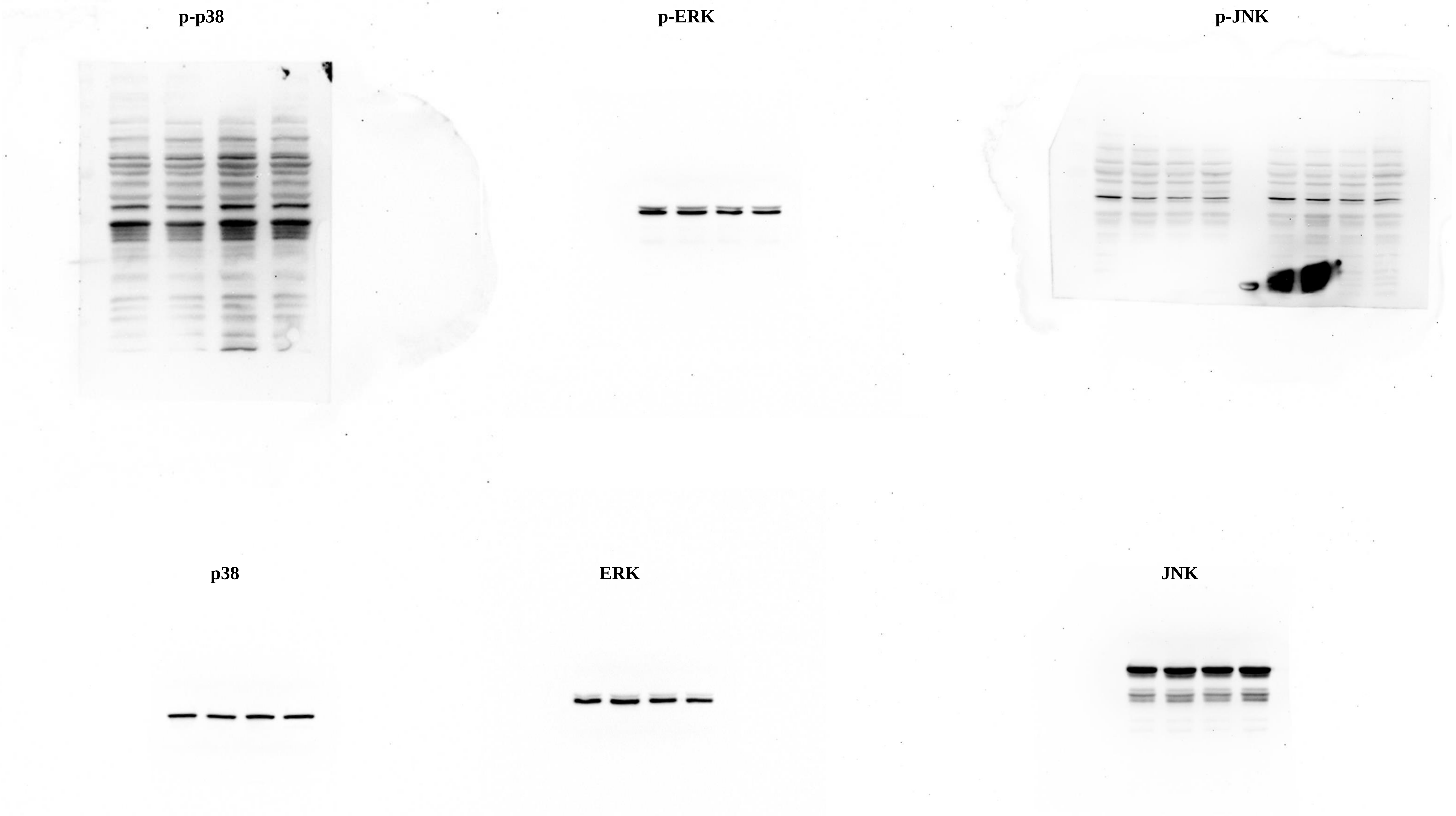

Figure 8.

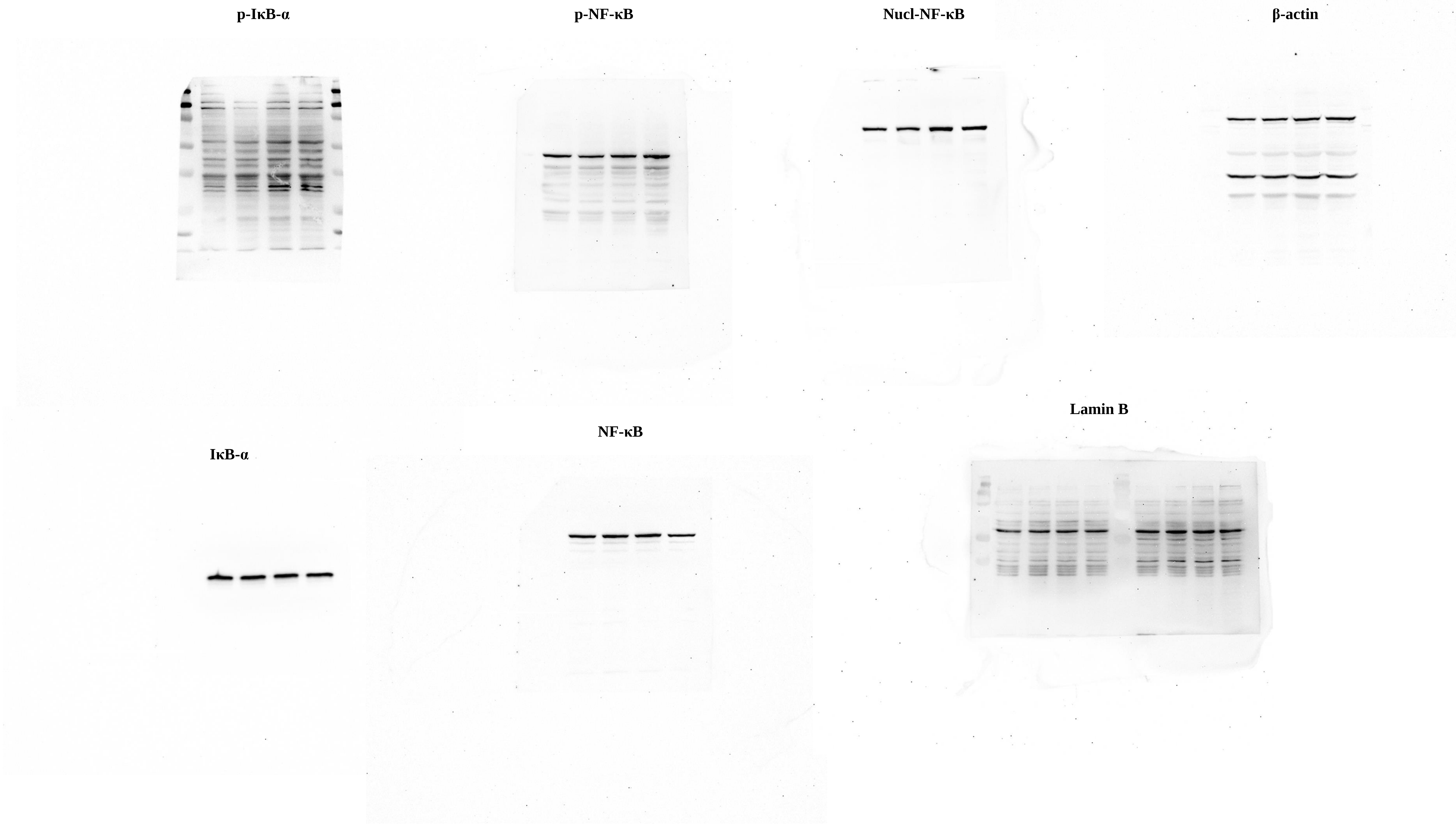

Figure 9.

p-p38

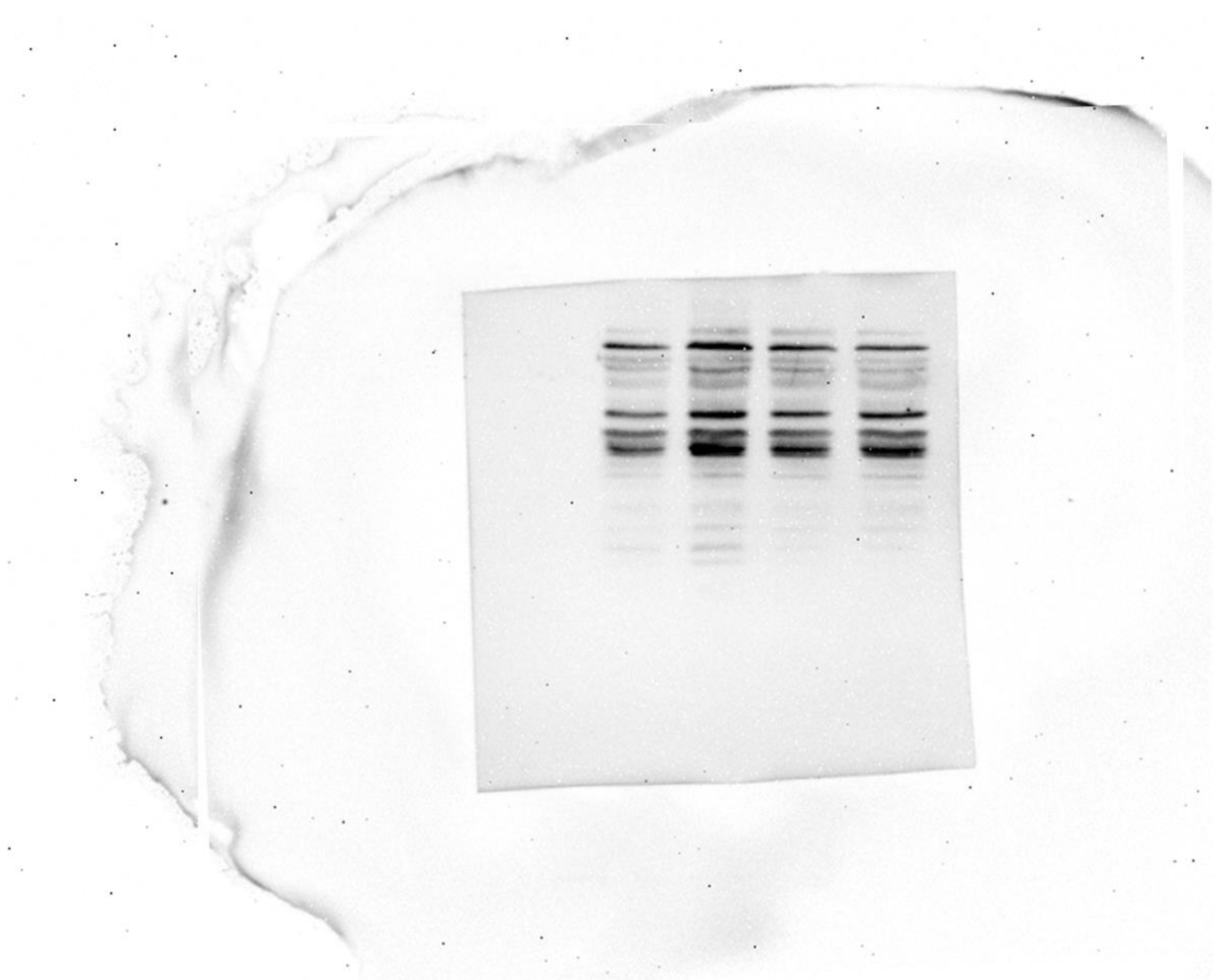

p-NF-κB

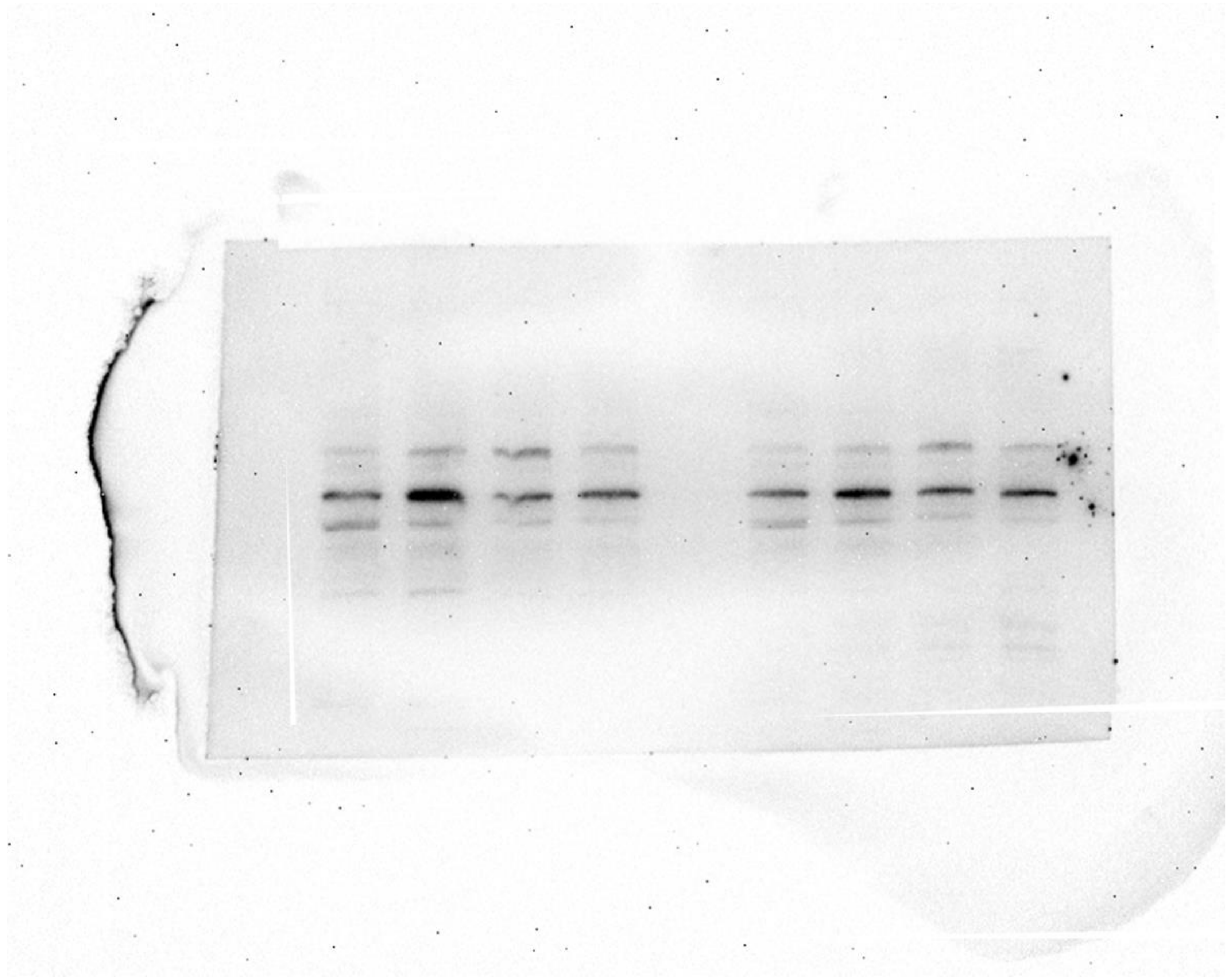

p38

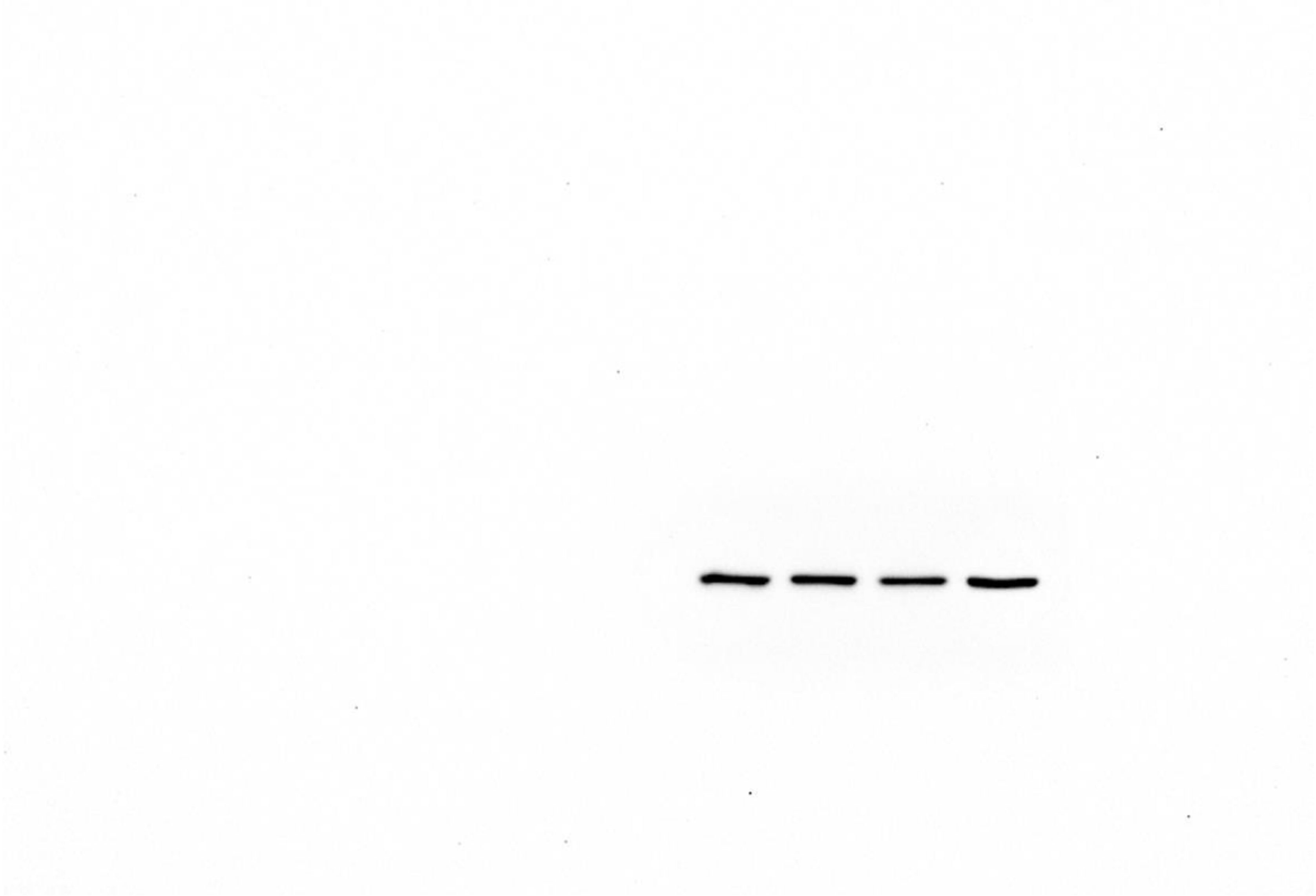

NF-κB

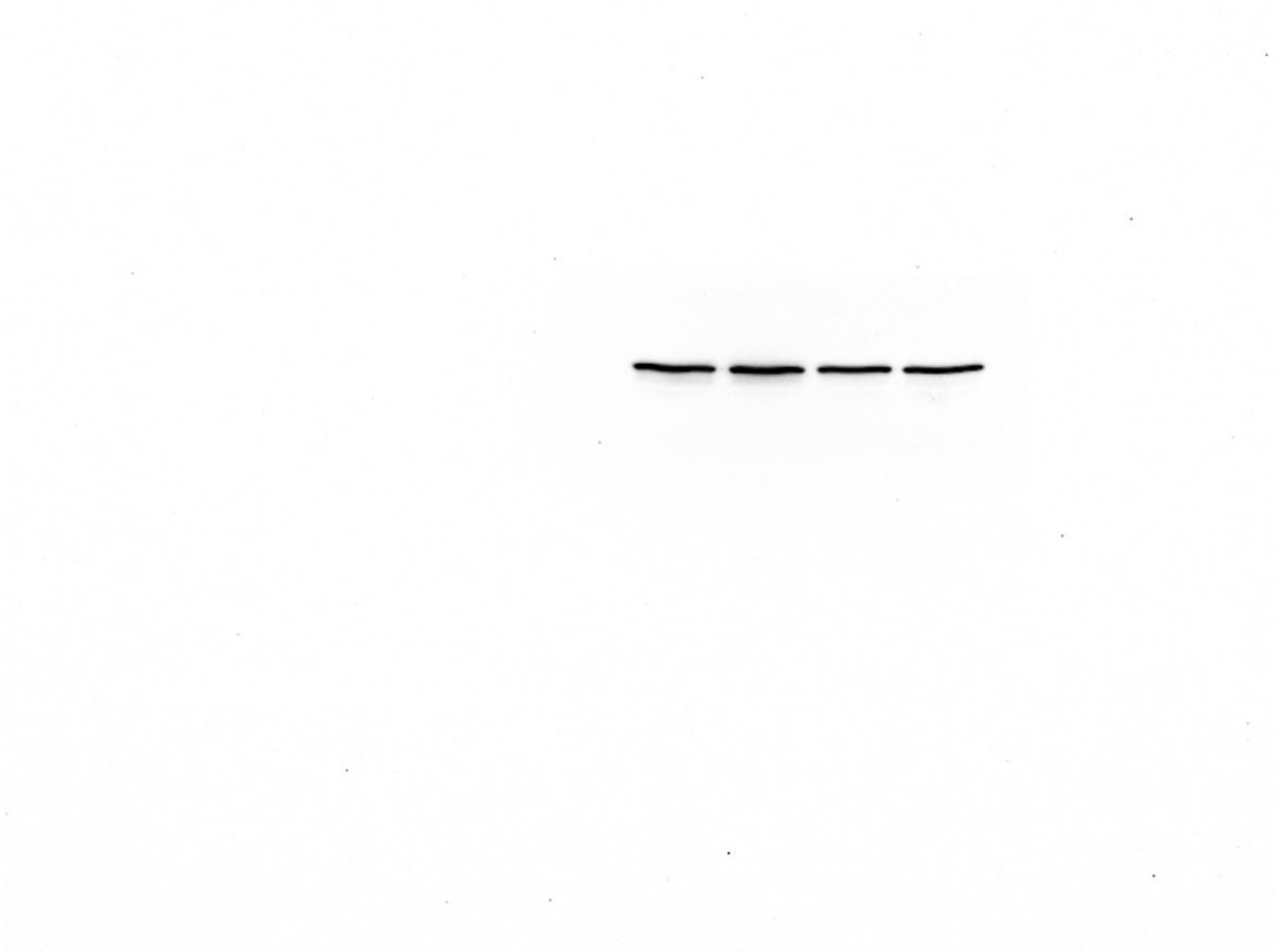

Figure 9.

TNF-α

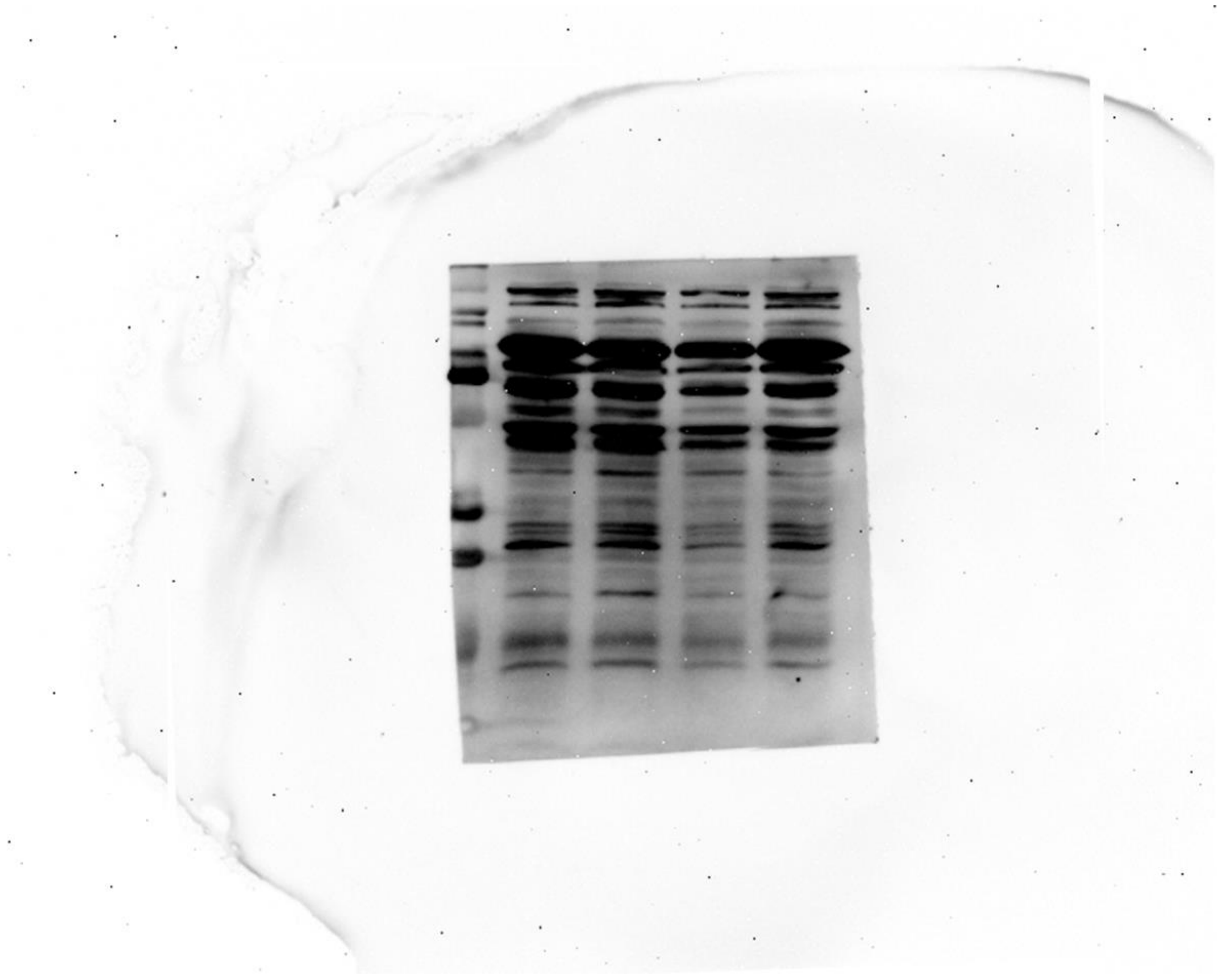

IL-6

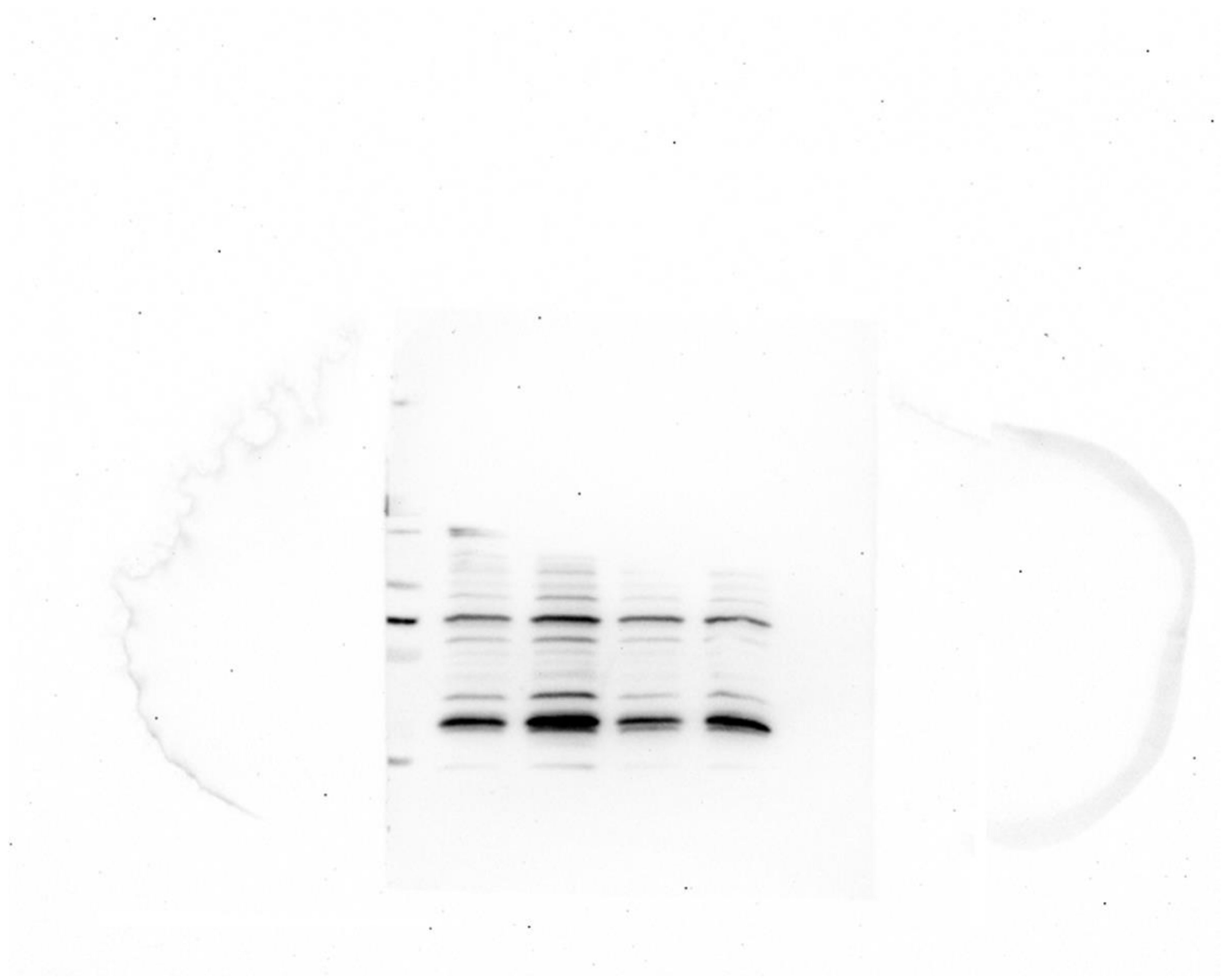

IL-1β

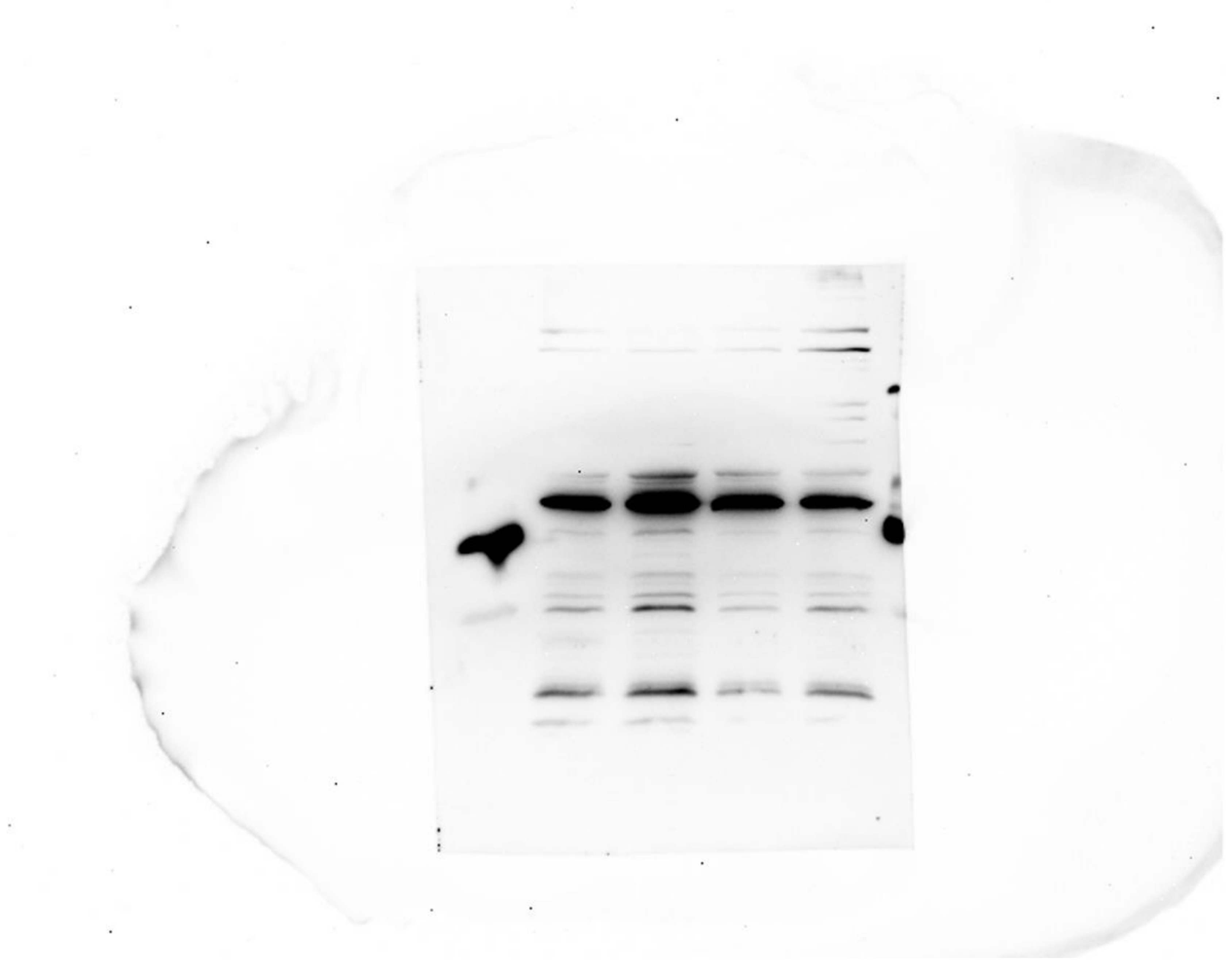

β-actin

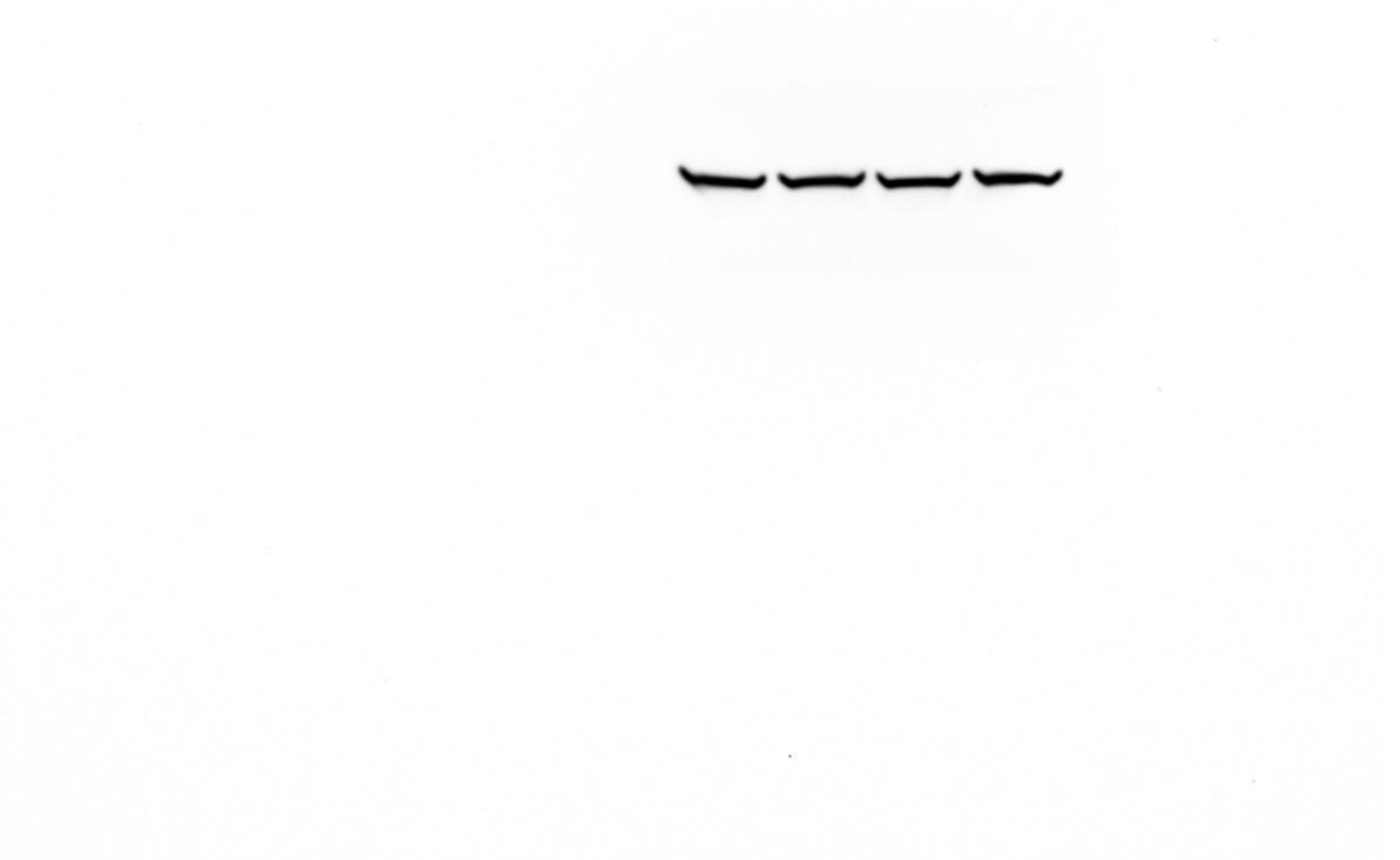

Supplementary 1.

CAT

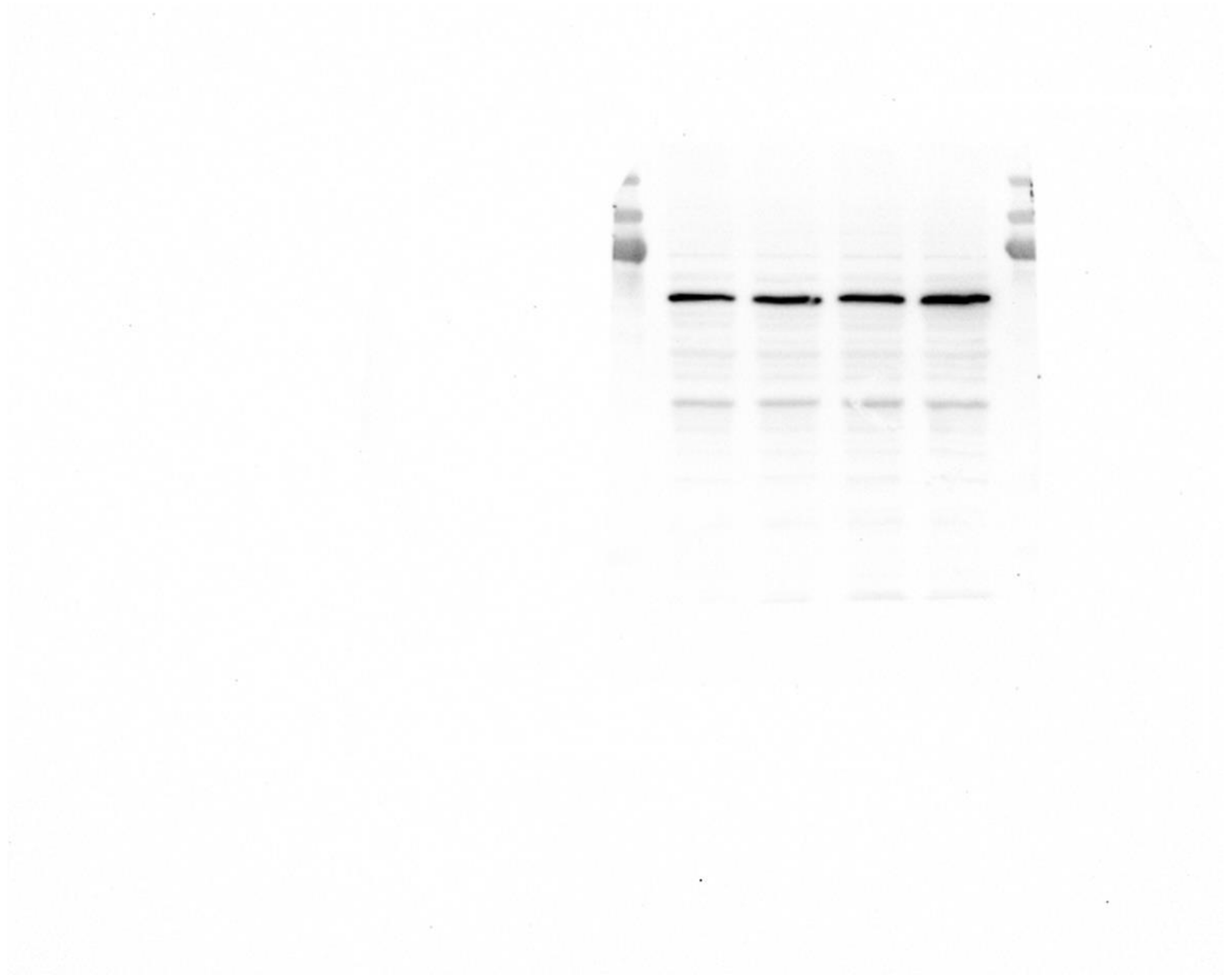

SOD1

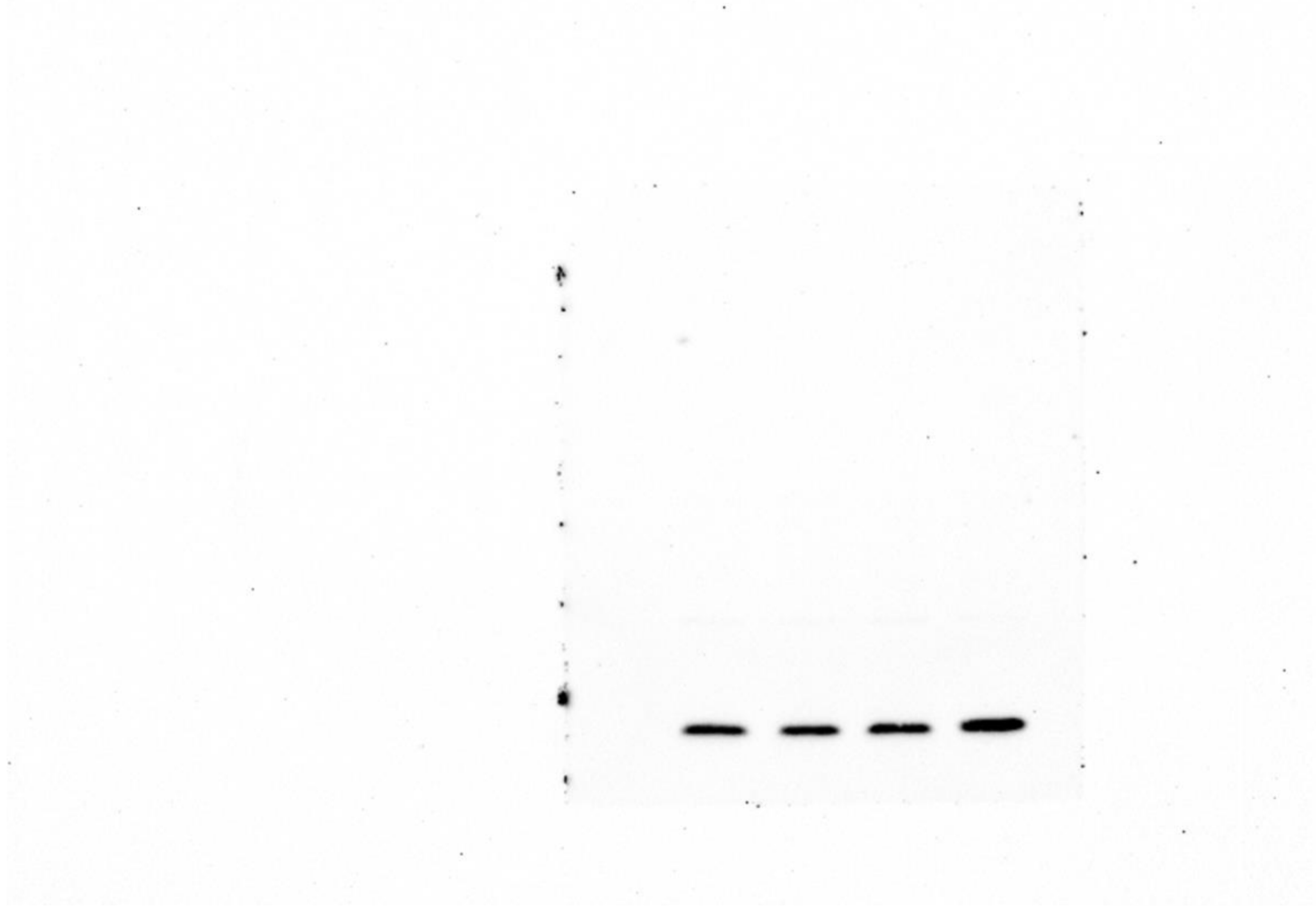

SOD2

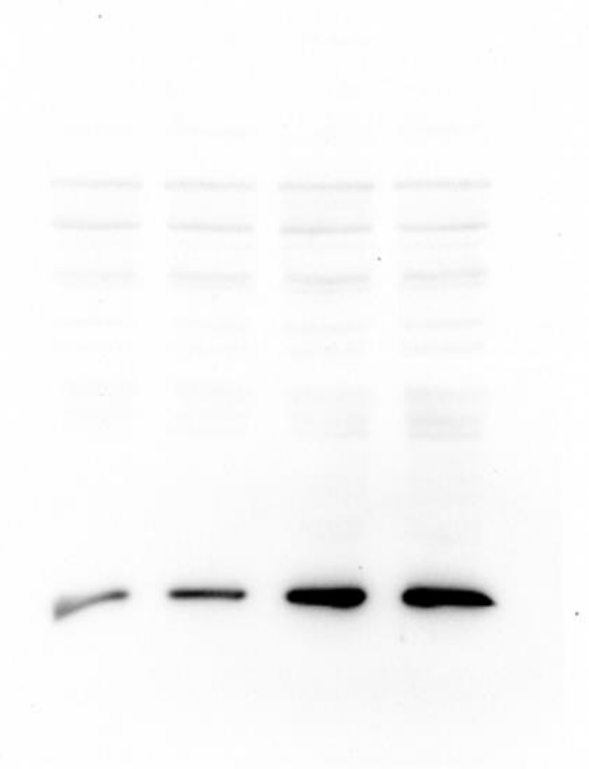

GPX1

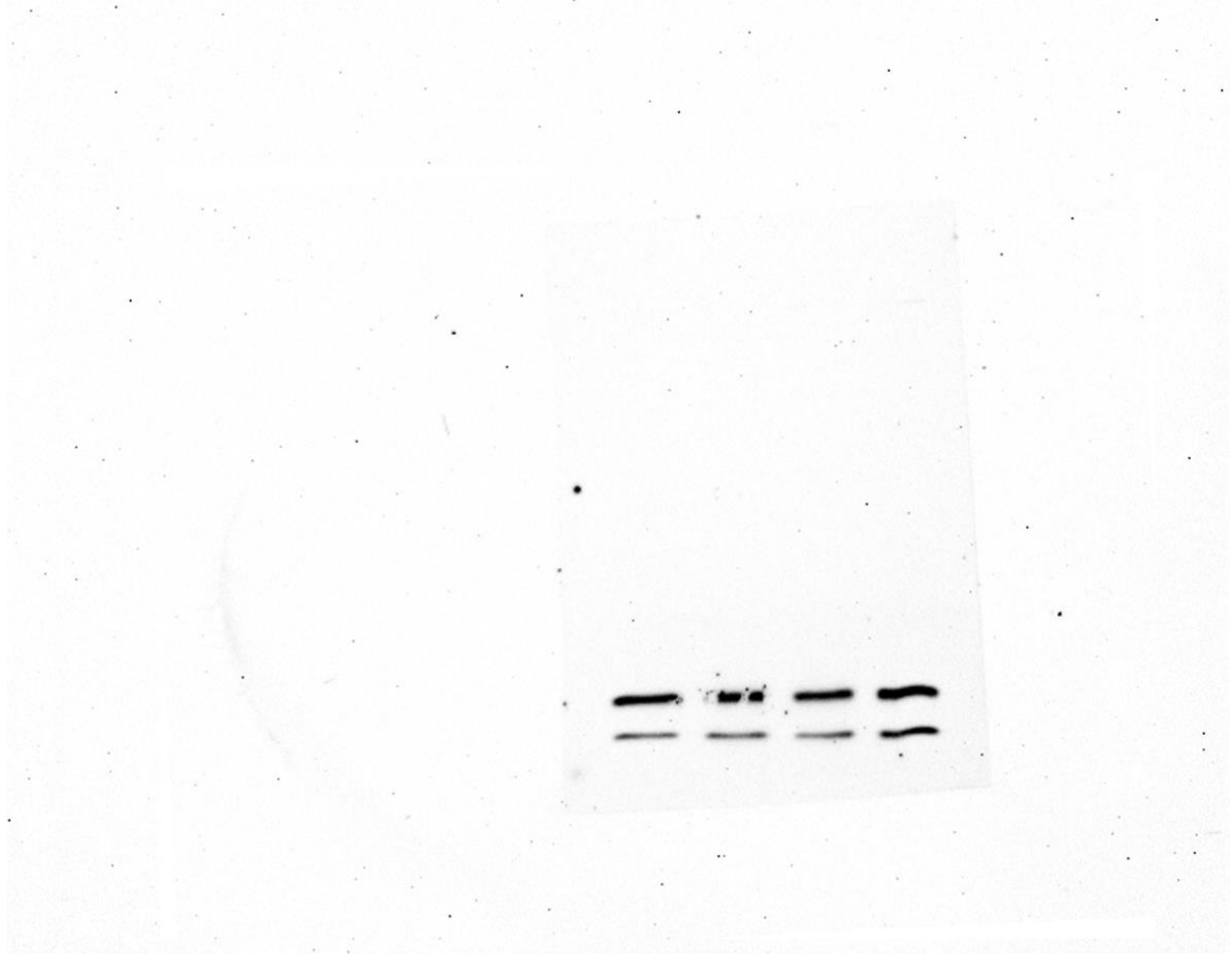

β-actin

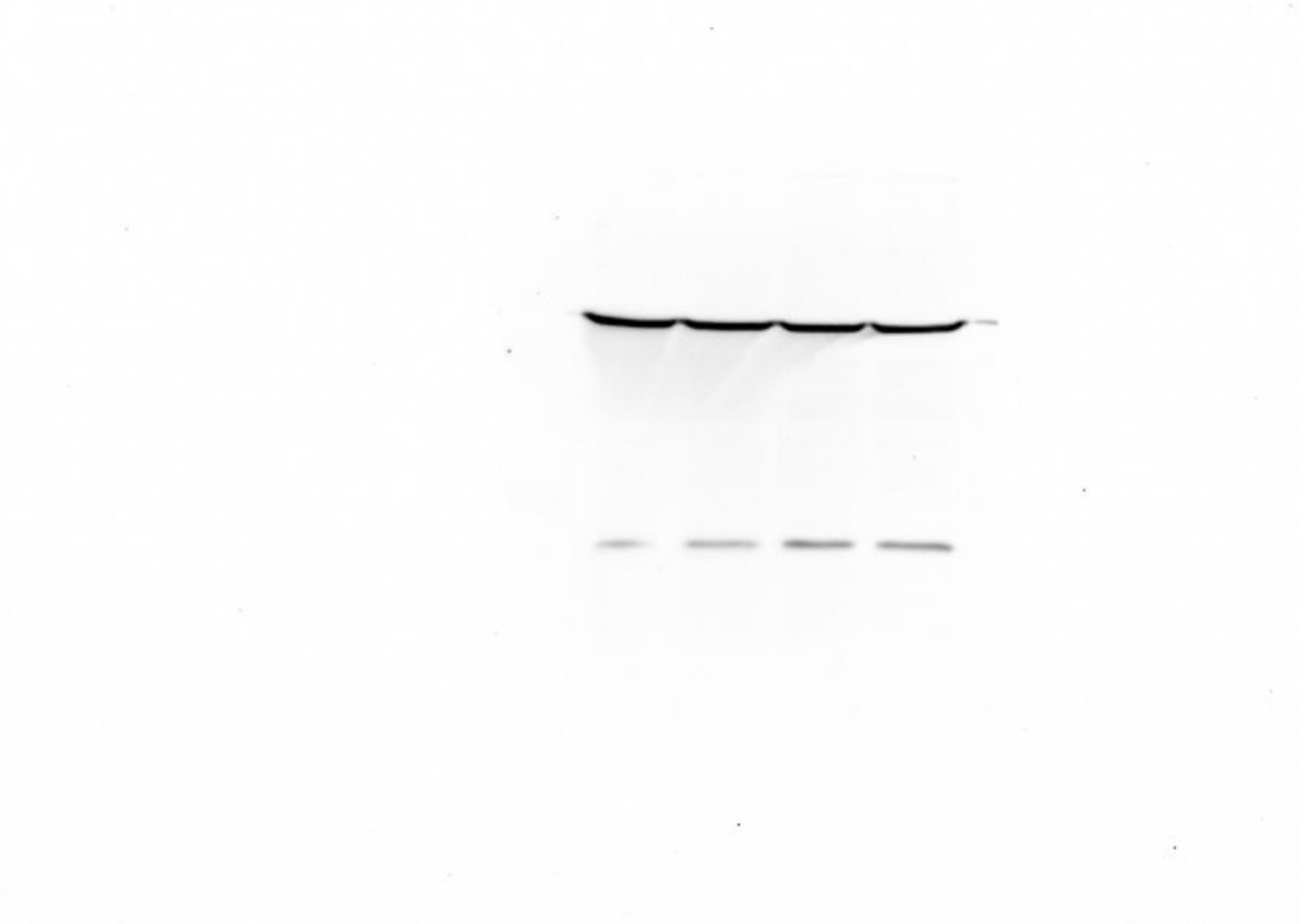

Supplementary 2.

BiP

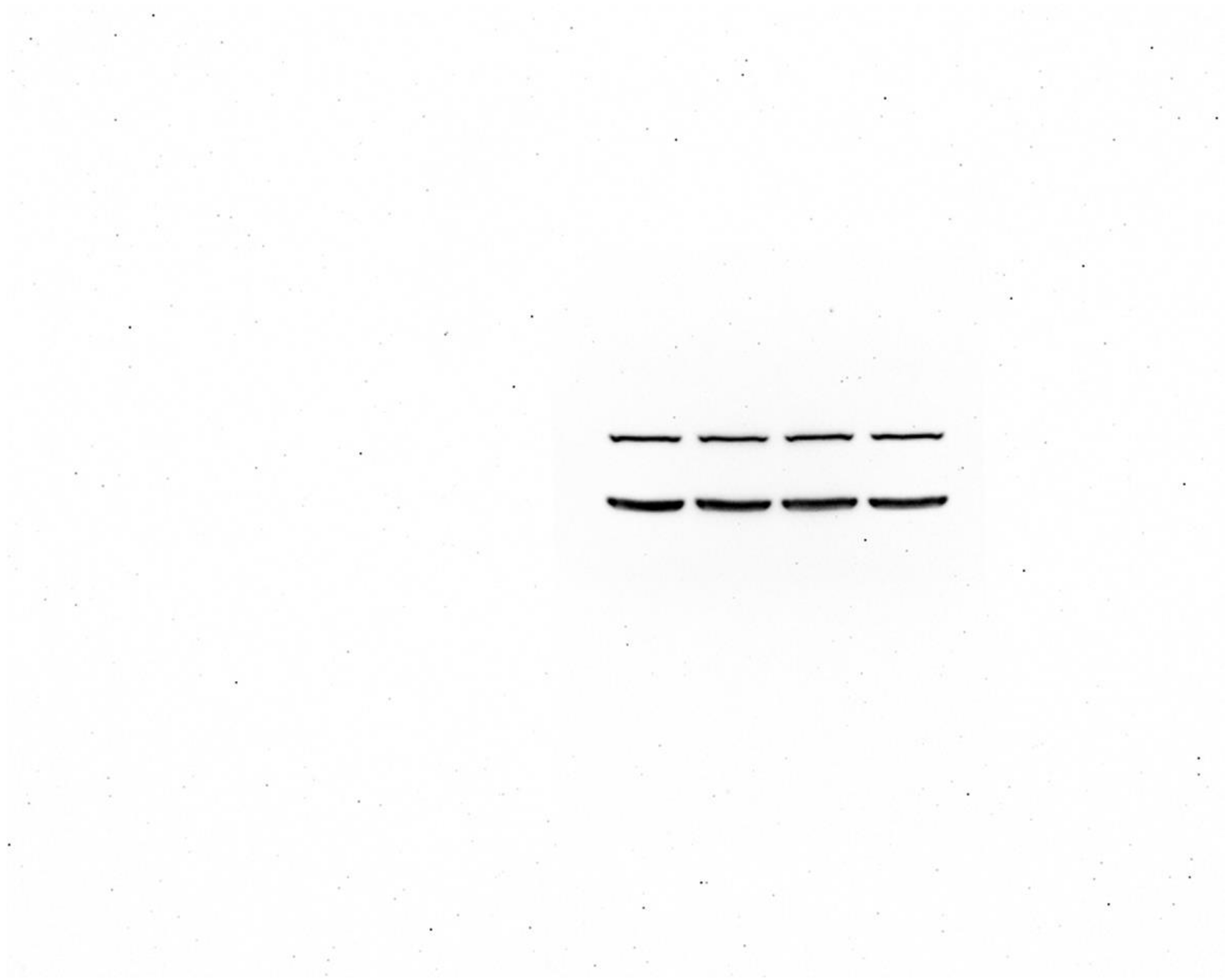

CHOP

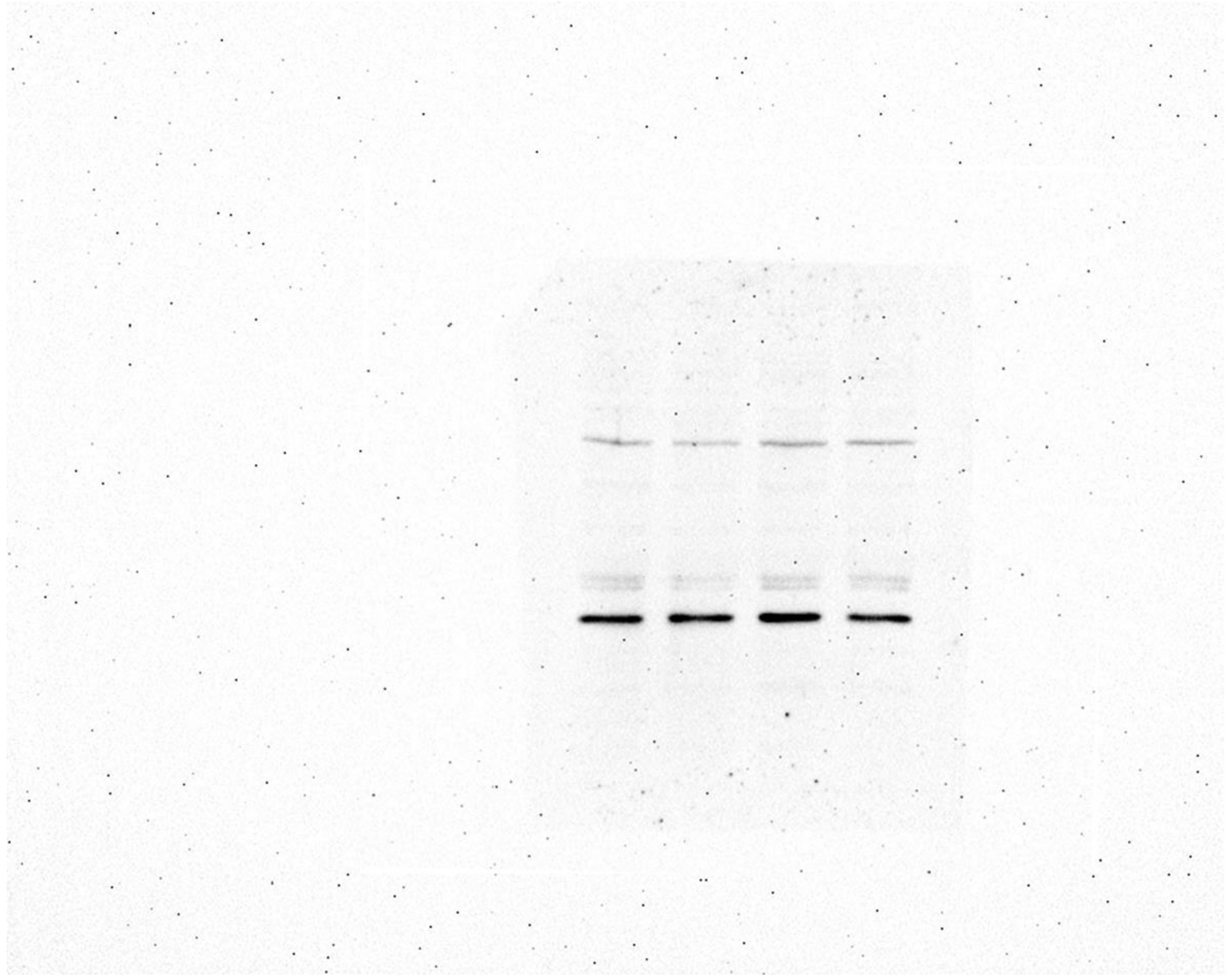

β-actin

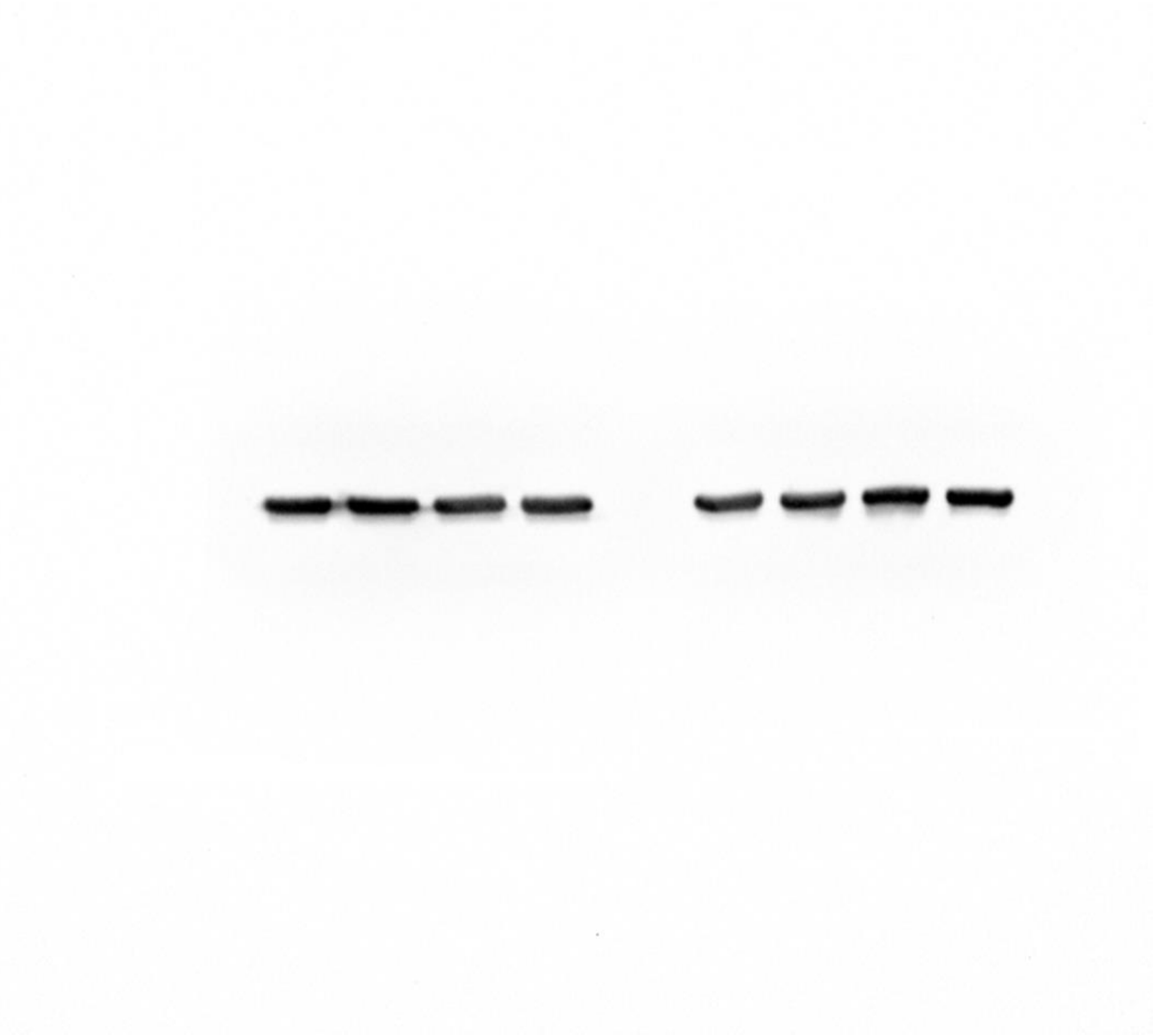

Supplementary 3.

Total-Nrf2

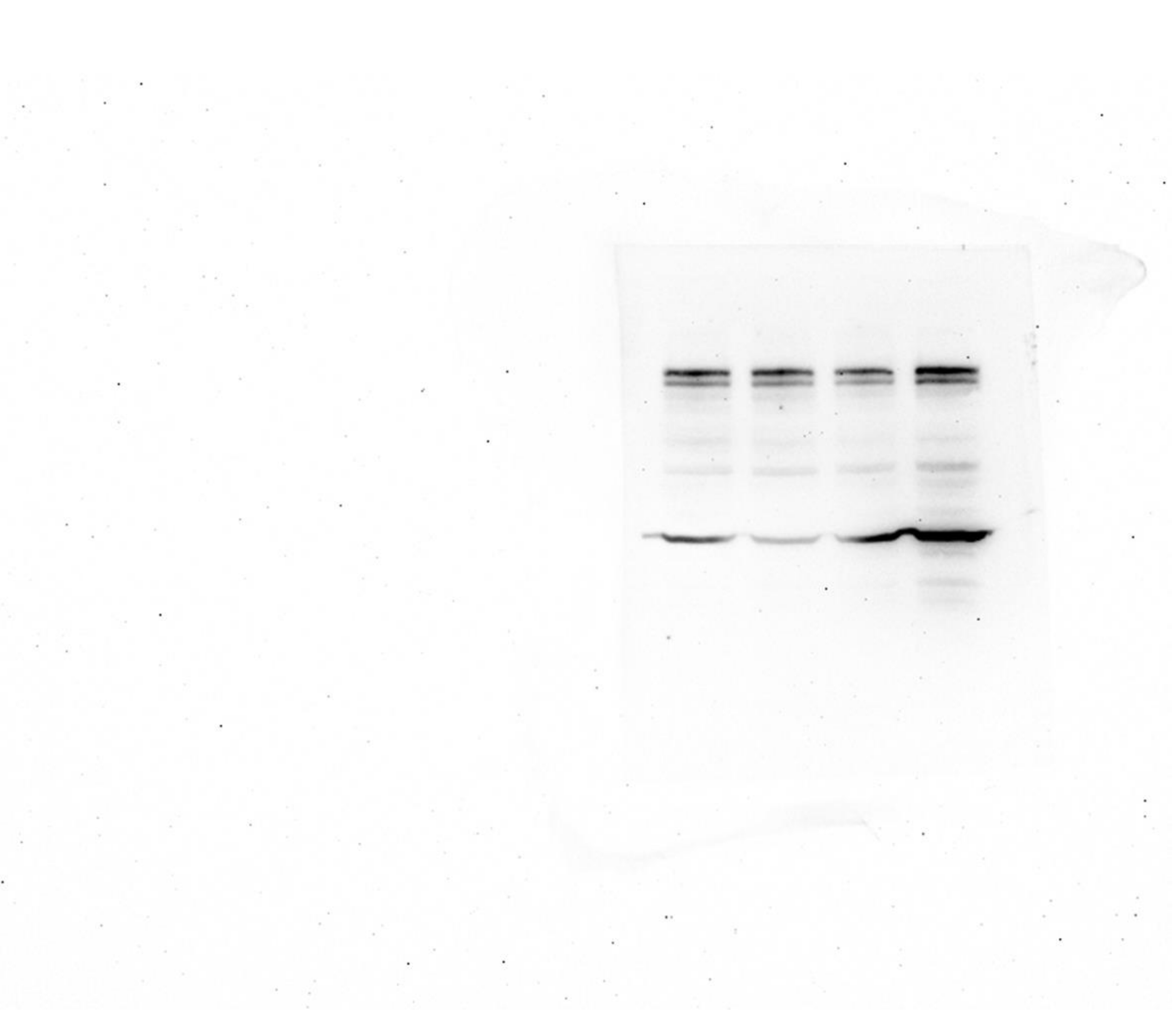

β-actin

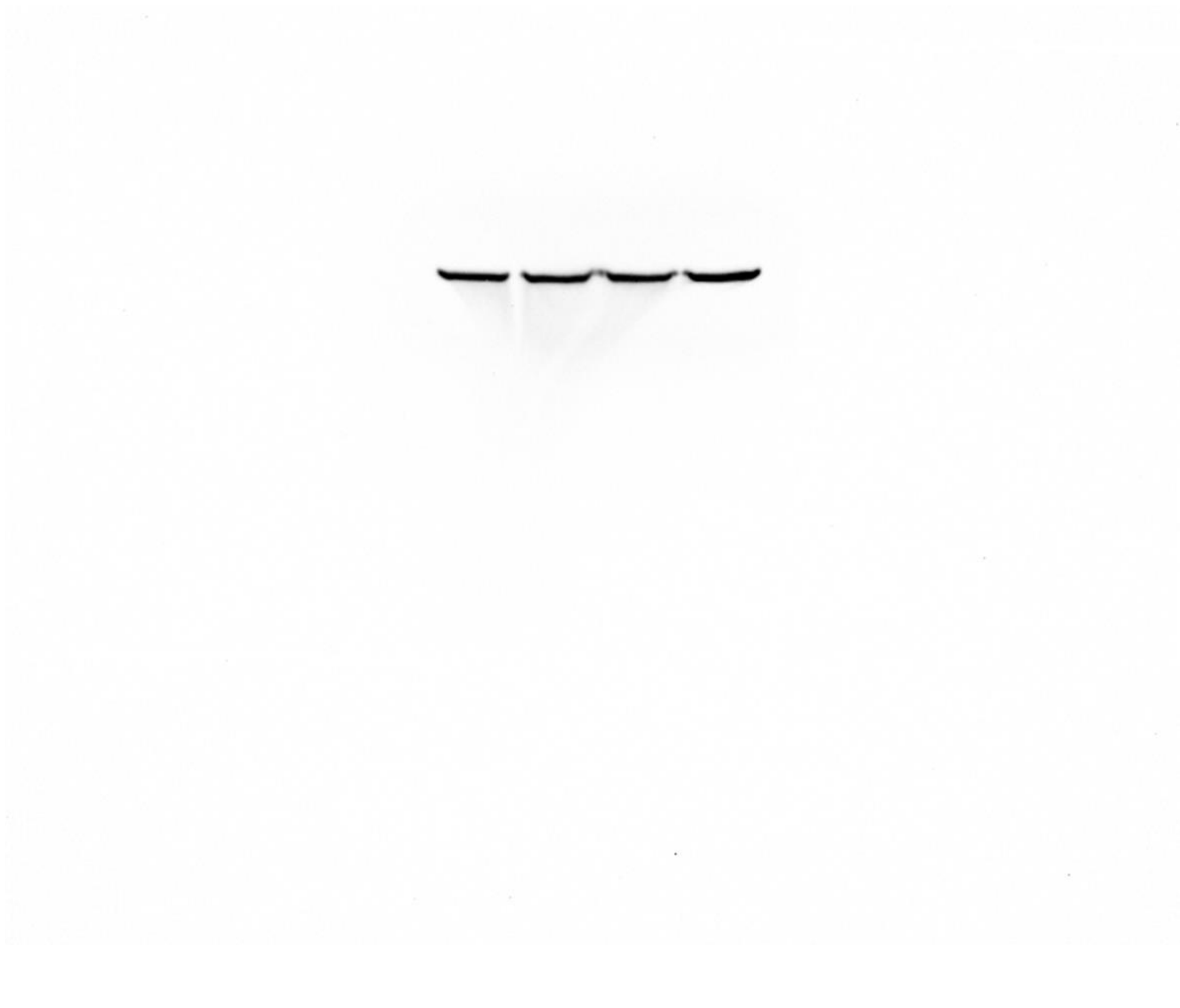

Nucl-Nrf2

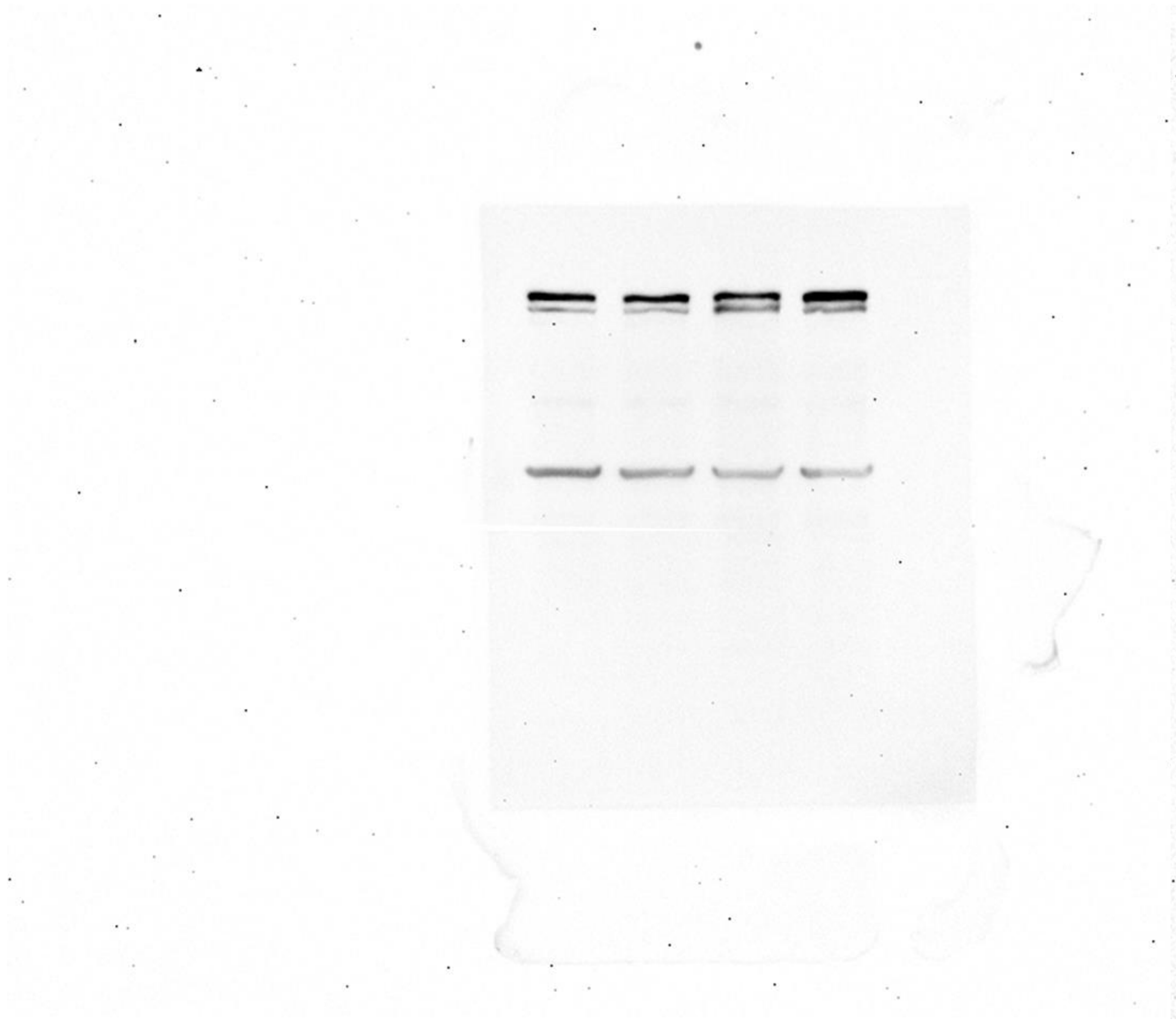

Lamin B

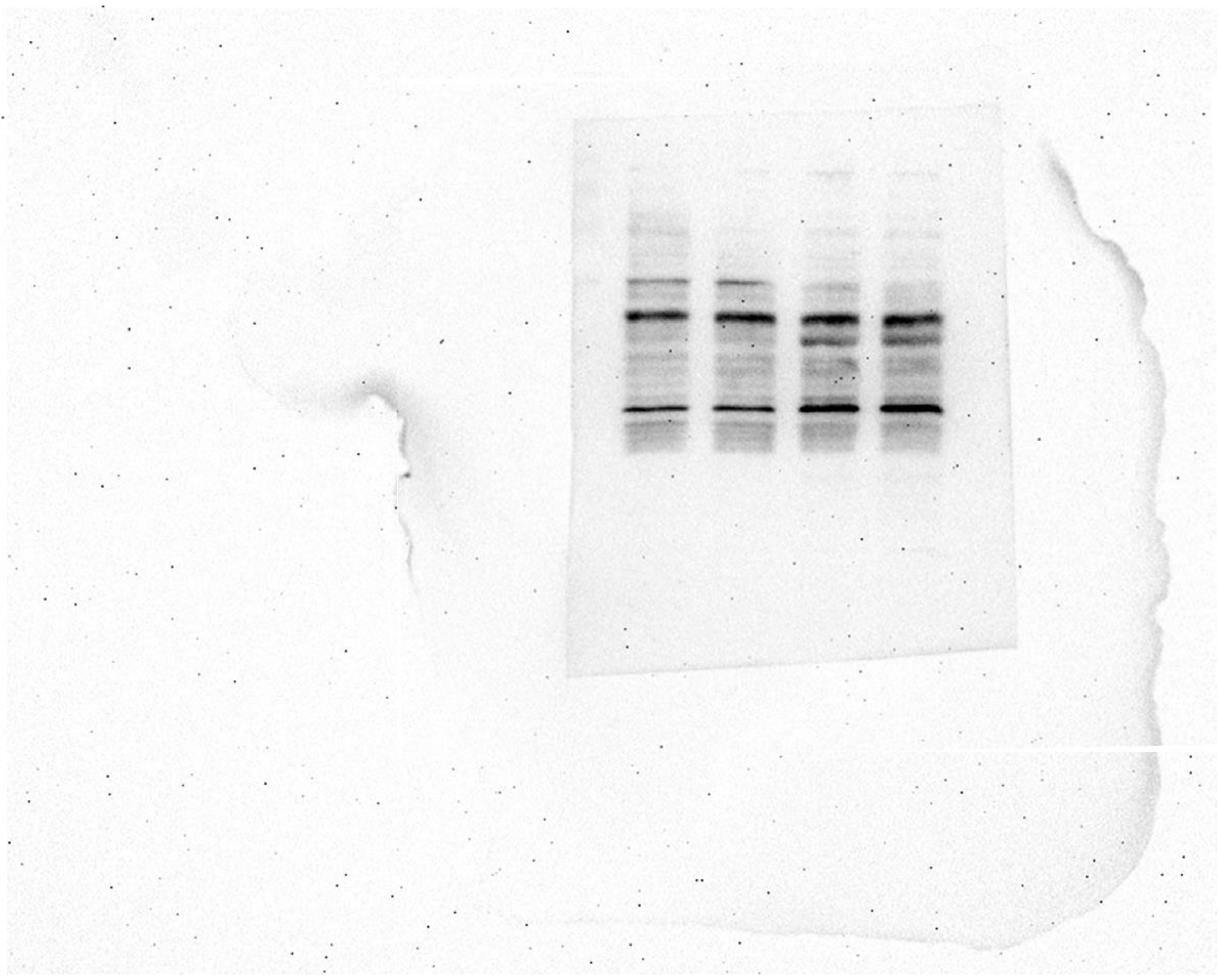

Supplement: Supplementary file 3 — Additional file 3. The original data for western blot results. [file 12989_2022_512_MOESM3_ESM.pdf]
